# Supplementary material for: Tracheal tuft cells release ATP and link innate to adaptive immunity in pneumonia
Source: Nat Commun. 2025 Jan 10;16:584. doi: 10.1038/s41467-025-55936-5 (PMC11724094; doi:10.1038/s41467-025-55936-5)
Supplement: Supplementary file 1 — Supplementary Information [file 41467_2025_55936_MOESM1_ESM.pdf]

Tracheal tuft cells release ATP and link innate to adaptive immunity in pneumonia

Noran Abdel Wadood[1#], Monika I. Hollenhorst[1,2#], Mohamed I. Elhawy[1#], Na Zhao[1], Clara Englisch[1], Saskia B. Evers[1], Mahana Sabachvili[1], Stephan Maxeiner[1,2], Amanda Wyatt[3], Christian Herr[4], Ann-Kathrin Burkhart[1,2,5], Elmar Krause[6], Daniela Yildiz[2,7], Anja Beckmann[1], Soumya Kusumakshi[3], Dieter Riethmacher[8], Markus Bischoff[9], Sandra Iden[1,2,5], Sören L. Becker[9], Brendan J. Canning[10], Veit Flockerzi[7], Thomas Gudermann[11,12], Vladimir Chubanov[11], Robert Bals[2,4,13], Carola Meier[1], Ulrich Boehm[2,3], Gabriela Krasteva-Christ[1,2*]

* Corresponding author

SUPPLEMENTARY METHODS:

ATP sensor experiments

For ATP-release experiments, mice were euthanized by inhalation of an overdose of the narcotic isoflurane (Piramal Critical Care Deutschland GmbH, Hallbergmoos, Germany) followed by cervical dislocation and aortic exsanguination. The explanted tracheas from *Trpm5*$^{+/+}$ and *Trpm5*$^{-/-}$ mice were separated into three pieces and incubated with 1 mM denatonium (Sigma-Aldrich, Taufkirchen, Germany) in 200 µl of buffer (136 mM NaCl (Grüssing GmbH, Filsum, Germany), 5.6 mM KCl (MERCK, Darmstadt, Germany), 10.7 mM glucose (Sigma-Aldrich), 10 mM HEPES (Carl Roth, Karlsruhe, Germany), 1 mM $MgCl_2$ (MERCK, Darmstadt, Germany), 2.2 mM $CaCl_2$ (Grüssing GmbH)) for 5 min at 37°C. For control experiments the tracheal pieces were incubated in 200 µl buffer solution without denatonium. Tracheas were then homogenized, centrifuged for 5 min at RT at 1500x g and the supernatants were frozen for ATP measurements. HEK293 cells (provided by VF, ATCC No. CRL-1573) were transiently transfected with an ATP-sensor (pm-iATPSnFR1.1) (Lobas *et al*, 2019) using Lipofectamine 2000 (Invitrogen, Thermo Fisher Scientific, Waltham, MA, USA) in Opti-MEM (Gibco, Thermo Fisher Scientific, Waltham, MA, USA) for 8 h according to the manufacturer's protocol. The pm-iATPSnFR1.1 plasmid was a gift from Baljit Khakh (Addgene plasmid #102549; http://n2t.net/addgene:102549; RRID: Addgene_102549). Cover slips seeded with transfected cells were incubated with Tyrode III buffer (130 mM NaCl, 10 mM HEPES, 10 mM glucose, 8 mM $CaCl_2$, 5 mM KCl, 10 mM sodium pyruvate (Gibco), 5 mM $NaHCO_3$ (Sigma-Aldrich) and 1 mM $MgCl_2$) and mounted under a microscope (Eclipse FN1, Nikon Instruments, Amsterdam, Netherlands). The changes in pm-iATPSnFR1.1 fluorescence were measured at 525 nm emission before and after application of tracheal supernatants. The light source and the shutter were controlled by a LAMBDA DG-4 control device containing a xenon arc lamp (Sutter Instruments, Novato, CA, USA). Acquired values

were processed using the NIS-Elements software AR 5.10.01 (Nikon Instruments, Amsterdam, Netherlands).

In a different set of experiments HEK-293 cells in a 6-well plate were transfected with the ATP-sensor plasmid (3 µg) with 9 µl Fugene HD (Roche, Mannheim, Germany) transfection reagent per well upon reaching 70% confluence. After 2 days, cells were resuspended with 0.05% trypsin and seeded into adherent epithelial cells for use. ATP-measurements were conducted simultaneously to whole cell patch clamp experiments as described below. The data are represented as F/F0. F0 refers to the fluorescence intensity of the recorded ATP sensor cells in the beginning (baseline in experiments performed without simultaneous patch clamp recordings or before whole-cell configuration of tuft cells in measurements with simultaneous patch clamp recordings). F refers to the fluorescence intensity of ATP sensor cells after stimulation at different time points or the maximum response depending on the respective experiment.

ATP measurements

The concentration of ATP in tracheal supernatants was assessed using a commercially available ELISA kit according to the manufacturer's instructions (product no. USC-CEA349GE-96, Cloud Clone Corp., Houston, TX, USA). Briefly, tracheas isolated from *Trpm5*$^{+/+}$, *Trpm5*$^{-/-}$ and *Panx1*$^{-/-}$ mice were stimulated with 1 mM denatonium (Sigma-Aldrich) or with 100 µM clozapine-N-oxide (CNO, HelloBio, Bristol, UK) for tracheas explanted from *Trpm5*-DREADD mice. Stimulation was performed after tracheal explantation in 200 µl of a solution containing 5.6 mM KCl, 136 mM NaCl, 10.7 mM glucose, 10 mM HEPES, 1 mM MgCl2 and 2.2 mM $CaCl_2$ for 5 min at 37°C. Supernatants used for ATP measurements were collected by centrifugation at 1000g for 5 min. For the ELISA assay, 50 µl of each sample was added to a well of a 96 well plate, followed by 50 µl of detection reagent A. After mixing, the plate was incubated for 1 h at 37°C and then washed 3 times with wash buffer. Next, 100 µl of detection reagent B were added, and the plate was incubated for 30 min at 37°C, followed by 5 washes.

Then, 90 µl of substrate solution were added, and the plate was incubated for approximately 20 min at 37°C. Finally, 50 µl of stop solution was added, and the plate was read at a wavelength of 450 nm. Analyses were performed using an EnSight Multimode Plate Reader (Perkin Elmer™, Rodgau-Jügesheim, Germany) with the software Kaleido 2.0 (Perkin Elmer).

ACh measurements

ACh measurements were performed using MALDI-TOF (Matrix-assisted laser desorption/ionization-time of flight) mass spectrometry. First, tracheal supernatants (1µl) were spotted onto the MALDI-TOF target plate (Bruker Daltonics, Billerica, MA, USA). Samples were measured in duplicates. The dried spots were overlaid with 1 µl of α-cyano-4 hydroxycinnamic acid (CHCA) matrix solution (Bruker Daltonics) consisting of saturated CHCA 50% (v/v) of acetonitrile, 2.5% (v/v) of trifluoroacetic acid and 47.5% (v/v) of LC-MS grade water. Calibration was performed using the Bacterial Test Standard (BTS) (Bruker Daltonics). After drying, the plate was placed into the Microflex LT Mass Spectrometer (Bruker Daltonics) for measurement. 240 laser shots in six random positions were carried out for each spot to generate the mass profiles in linear positive ion mode. For the measurement, a laser frequency of 60 Hz together with a high voltage of 20 kV were used to create mass charge ratio ranges (m/z) between 50 Da and 2 kDa. The raw spectra were obtained and analyzed using the FlexAnalysis® software version 3.4 (Bruker Daltonics) after withdrawing all flatlines and outlier peaks.

Immunohistochemistry

Immunohistochemistry experiments were performed as described previously (Hollenhorst *et al*, 2020). Briefly, tracheae from *ChAT*-eGFP, *Panx1*$^{-/-}$, *Trpm5*$^{-/-}$ or *Trpm5*$^{+/+}$ mice was fixed by transcardiac perfusion with Zamboni fixative consisting of 2% paraformaldehyde (PFA) and 15% saturated picric acid in 0.1 M phosphate buffer (pH 7.4) followed by three days of washing

in PBS (phosphate-buffered saline). For cryoprotection the tracheas were incubated overnight in sucrose (18%) dissolved in phosphate buffer (0.1 M), embedded in Tissue-Tek (Sakura Finetek, Torrance, CA, USA) and frozen in melting isopentane. Serial sectioning of 10 µm thick sections was performed on a cryostat (Leica CM 1950, Bensheim, Germany). To avoid repeated evaluation of cells (present in neighboring regions), the distance between two adjacent sections, which were subjected to evaluation, was 200 µm. Tracheal sections were incubated for 1 h at room temperature in a blocking solution consisting of 0.1% BSA (Biomol GmbH, Hamburg Germany), 0.5% Tween 20 (Sigma-Aldrich) and 10% horse serum (Gibco) with pH 7.4. Application of the primary antisera directed against pannexin 1 (1:200, rabbit, Alomone labs, ACC-234, TMH Medizinhandel, Duisburg, Germany), Trpm5 (1:800, rabbit, created in-house (Hollenhorst *et al*, 2020)), doublecortin-like kinase 1 (DCAMKL1, 1:1600, rabbit, ab31704, abcam, Cambridge, UK), ChAT (1:400, goat, AB144P, Sigma-Aldrich), Gnat3 (1:400, goat, pab73402, Covalab), GFP (1:2000, chicken, NB100-1614, Novusbio) and CD11c (1:800, Armenian hamster, 14-0114-82, eBioscience, San Diego, CA, USA) was performed overnight at room temperature followed by three washing steps with PBS for 10 min each. Tissue was then incubated with the secondary antibody conjugated to Cy3 (donkey anti-rabbit-Cy3, Merck Millipore, AP182C, 1:1000, donkey anti-goat-Cy3 Millipore, AP180C, 1:250, or goat anti Armenian hamster, Jackson, 127-165-160, 1:800) or to Cy2 (donkey anti-rabbit-Cy2, Jackson, 711-225-152, 1:100) or to AF647 (donkey anti-goat-AF647, Millipore, AP180SA6, 1:400), or to FITC (donkey anti-chicken-FITC, Jackson Immuno Research, 800-367-5296, 1:200) for an hour at room temperature. Tissue sections were washed once with PBS, incubated with DAPI (4´,6-diamidino-2-phenylindole, Carl Roth) for 10 min, washed again in PBS, postfixed with 4% PFA for 10 min and mounted with Mowiol 4-88 (Carl Roth, Karlsruhe, Germany) under a coverslip. Negative controls were performed by omitting the primary antibody and incubating solely with the secondary antibody.

To demonstrate co-localization of the TC marker Trpm5 together with beta-galactosidase (lacZ) by immunohistochemical means, bright field documentation was essentially performed as published previously using lacZ histochemistry in combination with immunohistochemistry (Maxeiner *et al*, 2003; Degen *et al*, 2004). Tracheas were dissected from transcardially Zamboni-fixed Panx1, Panx2, and Panx1/2 reporter mice (Bargiotas *et al*, 2011). Tracheas were washed, cryo-protected and sectioned at 10 µm, collected on cryo-slides and air-dried for 1 hr. Subsequently, slides were washed twice for 10 mins in lacZ washing buffer (cf. all staining solutions to (Degen *et al*, 2004). After removal of the washing buffer, slides were incubated in lacZ-staining solution at 37°C overnight to allow for the proceeding of beta-galactosidase enzyme activity. On the following day, successful staining was determined by inspecting slides for the presence of positive blue precipitates resulting from the enzymatic catalysis of X-Gal (Carl Roth, Karlsruhe, Germany) by beta-galactosidase. Slides were washed twice briefly for 5 min in PBS buffer before switching to the workflow regarding the immunodetection of Trpm5. LacZ-positive slides were incubated for 1 h in a solution of 10% methanol, 90% PBS and 0,1% hydrogen peroxide to inactivate endogenous peroxide activity. Slides were rinsed twice for 5 min in PBS, before sections were incubated in blocking solution (see above) to block unspecific protein binding sites. After removal of the blocking reagent, sections were incubated over night with the primary antibody diluted in PBS (rabbit anti-Trpm5 794, affinity-purified), see above, (Hollenhorst *et al*, 2020). On the third day, the incubation with a biotinylated secondary antibody, the incubation in ABC-reagent (streptavidine/peroxidase-based immunohistochemistry, Vectastain® ABC-Kit, PK-4001) as well as the incubation in the substrate solution (Vector® NovaRED™ peroxidase substrate solution, SK-4800) were all performed according to the manufacturer’s protocol (Vector Laboratories, Newark, CA, USA, distributed by Linaris GmbH, Dossenheim, Germany). Finally, sections were mounted under coverslips using Kaiser’s glycerol gelatine solution (Merck, Darmstadt, Germany). The

evaluation of all immunofluorescence and histochemical stainings was performed using an AXIO imager M2 microscope with the Zen pro v2.3 software (Zeiss, Oberkochen, Germany).

Patch-clamp experiments

The tracheas from *ChAT*-eGFP or *Trpm5*$^{-/-}$-*ChAT*-eGFP mice were dissected from mice euthanized with an overdose of ketamine (Zoetis, Berlin, Germany) / xylazine (Bayer, Leverkusen, Germany) *i.p.* and separated into small pieces. The pieces were then transferred to Ca^{2+} free Tyrode solution (140 mM NaCl, 5 mM KCl, 10 mM HEPES, 10 mM glucose, 1mM $MgCl_2$ and 2.5 mM $CaCl_2$) supplemented with 2 mg/ml papain (P4762), 5 mM L-Cysteine (C7352), 150 U/µL DNase I (18047019) (all reagents were purchased from Sigma-Aldrich) and 1 mM EDTA (Grüssing GmbH) at 37 °C for 30 min. This digestion solution was further diluted into DMEM (11965092, Gibco, Thermo-Fisher) and centrifuged at 250x g for 5 min. The supernatant was aspirated, and the cell pellet was resuspended in DMEM and then seeded onto 25 mm coverslips coated with Cell-Tak (Corning™, Thermo-Fisher, prepared according to the manufacturer's protocol). Cells were allowed to adhere for at least 1h before use.

Coverslips were transferred to the recording chamber and continuously perfused with Tyrode solution as described above. GFP^{+} TCs were patched under a Nikon microscope (TE2000e, Tokyo, Japan) with a 40x water immersion objective and filter sets for GFP excited by a LED illumination lamp (CoolLED, Andover, UK). Images were captured with an ORCA-spark Digital CMOS camera (Hamamatsu Photonics, Hamamatsu, Japan). Whole-cell membrane currents were recorded using an EPC 10 USB amplifier (HEKA Elektronik GmbH, Lambrecht, Germany) low pass filtered at 3 kHz and data acquisition was controlled by the Patchmaster software version 2x90.3 (HEKA). Patch pipettes (resistance, 4–5 MΩ) were pulled from borosilicate capillaries (Outside diameter: 1.5 mm, inside diameter: 1.05 mm; Science Products, Hofheim am Taunus, Germany) using a DMZ universal electrode Puller (Zeitz-Instrumente, Planegg, Germany). Patch pipettes were filled with an intracellular solution containing 140 mM

CsCl, 2 mM $MgCl_2$, 10 mM HEPES and 2 mM Na_2ATP (pH~7.2). 10 mM EGTA and 1.55 mM $CaCl_2$ were added into the intracellular solution to achieve 110 nM free Ca^{2+}; 2 mM EGTA and 0.9 mM $CaCl_2$ were included to obtain 1.0 µM free Ca^{2+}; and 5 mM EGTA and 4.6 mM $CaCl_2$ were applied to reach 2.4 µM free Ca^{2+}. The holding potential in voltage-clamp mode was -60 mV. After establishing the whole-cell configuration, a voltage ramp protocol from -120 mV to +120 mV for 300 ms was applied every 5 second when currents were stable. The Trpm5 antagonist TPPO (100 µM) was focally applied to confirm Trpm5 currents. 60 mM KCl was used to activate pannexin-1 channels by hyperpolarization to -60 mV at the holding potential of +30 mV. To investigate the role of P_2X_7, AZ 10606121 (20 µM) was applied to high intracellular Ca^{2+} (1 µM) induced currents. To exclude effects of the cell size on the measured currents, we calculated the current density [pA/pF] for the data represented in Fig. 1A and Supplementary Fig. 1A as well as in Fig. 2 C and H. Current density was calculated by dividing the currents measured by the capacitance of the respective cell. Data were analyzed with the software Igor Pro 8.0.4.2 (WaveMetrics, Portland, USA).

Ussing chamber experiments

Ussing chamber experiments were performed essentially as described previously (Kumar *et al*, 2020). Briefly, mice were euthanized by inhalation of an overdose of isoflurane followed by cervical dislocation and aortic exsanguination. Then the trachea was dissected, cut longitudinally and mounted onto a modified Ussing chamber where it was perfused with a buffer solution (145 mM NaCl, 1.3 mM Ca^{2+} gluconate (Sigma-Aldrich), 1 mM $MgCl_2$, 5 HEPES, 5 mM glucose and 2 mM KH_2PO_4 (MERCK) with a pH adjusted to 7.4) and heated to 37°C in a water bath. For measuring the short circuit current (I_{SC}) we used a voltage clamp amplifier (KU Leuven, Belgium) connected via the analog-to-digital converter PowerLab version 4/35 (ADInstruments) to a computer with LabChart version 8 software (ADInstruments, Spechbach, Germany). Viability of the tissue was verified by application of

ATP (100 µM, apical, Sigma-Aldrich) concluding each experiment. Denatonium (1 mM) was used to stimulate TCs. Suramin (100 µM) was used to inhibit purinergic receptors and investigate paracrine action of released ATP. Probenecid (200 µM) was used to inhibit pannexin 1 channels. Ruthenium red (20 µM) was used as a blocker for Calhm1 channels.

Immunoblot Analysis

Sample preparation as well as immunoblot analysis were performed as has been reported previously (Hollenhorst et al. 2023). Briefly, tracheas were placed in 200 µl RIPA-buffer complemented with a protease inhibitor cocktail (cOmplete®, Roche Diagnostics, Mannheim, Germany) on a bacterial culture dish on ice. A rubber spatula was used to remove in four consecutive strokes the tracheal epithelium, epithelial debris on the face of the rubber spatula as well as in the preparative solution was collected (approx. 60 µl total volume). Protein quantification was performed using the BCA-method using the PierceTM BCA Protein Assay Kit (ThermoScientific, Rockford, IL, USA). A total amount of 40 µg protein of each sample was loaded on a 10% SDS-polyacrylamide gel. Samples were separated and, subsequently, transferred by blotting onto a nitrocellulose membrane. Unspecific protein binding sites on the membrane were saturated using a solution of 5% milk powder in TBS-T-buffer for 1 h. Incubation with the primary ChAT antibody (diluted 1:500; rabbit monoclonal antibody 13H9L16, Merck, Darmstadt, Germany) was performed over night at room temperature diluted in blocking buffer. On the subsequent day, the membrane was washed three times for 10 min with TBS-T-buffer before the horseradish peroxidase-coupled secondary antibody (anti-rabbit IgG; concentration, 1:10,000; Sigma-Aldrich, Steinheim, Germany) was applied in blocking buffer for 1 h. After three washing steps with TBS-T-buffer for 10 min, respectively, enhanced chemiluminescent reaction was detected upon application of reagents of the Supersignal West Pico Plus ECL Kit (Thermo Scientific). For Actin staining, the membrane was stripped and

incubated with a primary Actin antibody (diluted 1:200; rabbit polyclonal, Sigma-Aldrich, Merck, Darmstadt, Germany). Then the blot was processed as described above for secondary antibody incubation and development. Images of the blot were acquired with the software Image Lab version 4.0.1 (Bio-Rad, Feldkirchen, Germany) using a ChemiDoc XRS+ system (Bio-Rad).

Ca^{2+}-imaging experiments

Ca^{2+}-imaging experiments of isolated epithelial cells from Trpm5-DREADD-tGFP mice was performed as described previously (*4, 5, 23*). Briefly, epithelial cells were digested as described above for patch clamp experiments and loaded with Fura-2. TCs were stimulated with CNO (60 µM). The same set-up and software as described for the ATP sensor experiments was used.

In vivo tuft cell stimulation

Mice were anesthetized via intraperitoneal injection of ketamine/xylazine (90-120 mg/kg / 6-8 mg/kg; Zoetis/Bayer). After performing a minimally invasive surgery, the upper part of the trachea was exposed and either vehicle or 1 mM denatonium (Sigma-Aldrich) or 100 µM CNO (HelloBio) were applied through the median cricothyroid ligament using a 22-gauge needle (Hamilton, Reno, NV, USA). Additionally, all animals received analgesia with 100 µl Carprofen (*i.p.*, 1 mg/ml, Zoetis) at the end of the surgical procedure. To assess TC numbers and DC recruitment at different time points the animals were randomly assigned to 10 experimental groups. These were a control group without any treatment, a vehicle group with inhalation of PBS, experimental groups 24 h, 72 h and 7 days after denatonium inhalation. At the end of each experiment, animals were sacrificed 24 h, 72 h or 7 days after denatonium inhalation by an overdose of ketamine/xylazine. For immunohistochemistry experiments, mice were transcardially perfused with a solution containing 2,5% polyvinylpyrrolidone followed by Zamboni fixative (see above). Then, the middle part of the tracheas (between the 4th and the 9th

tracheal cartilages) and the lungs (*in toto*) were dissected and incubated with the fixative for one hour or for 12 hours, respectively. Afterwards the tissue was washed over the course of three days in 0.1 M phosphate buffer, and immunohistochemistry was performed as described above. For FACS experiments, organs were collected without prior perfusion (see below) 30 min or 72 h after the surgery.

Infection experiments with *P. aeruginosa*

Mice were infected with the mucoid *P. aeruginosa* strain NH57388A (provided by Niels Hoiby, Department of Clinical Microbiology, Rigshospialet, University of Copenhagen, Denmark) as described previously (Hollenhorst *et al*, 2022). Briefly, an inoculum of the bacterial strain was applied intratracheally under anesthesia with ketamine/xylazine (90-120 mg/kg / 6-8 mg/kg; Zoetis/Bayer). A maximum volume of 40 µl bacterial suspension was applied. The mice were sacrificed three days post infection. In total, 10 independent experiments were performed with inocula of 1-3*10^6 CFU. At the end of each experiment the animals were euthanized by an overdose of ketamine/xylazine (Zoetis/Bayer), the organs were removed and further processed for FACS analyses (see below). Mice were closely monitored during the whole experiment and *Trpm5*$^{-/-}$ as well as *Panx1*$^{-/-}$ mice reached the termination criteria more often than *Trpm5*$^{+/+}$ mice and therefore were euthanized upon reaching the criteria.

FACS analyses

Lungs, tracheae, bronchoalveolar lavage fluid (BALF) and airways lymph nodes from CNO or denatonium treated or *P. aeruginosa* infected mice or respective untreated control mice of each mouse strain that was used were collected and processed as described previously (Hollenhorst *et al*, 2022). Briefly, after the mice were euthanized (see above) blood samples were collected and afterwards BALF was obtained by instillation and aspiration of 1 ml of ice-cold DPBS with 0.1 mM EDTA and 1% fetal bovine serum (FBS) through a tracheal cannula into the lungs.

This step was repeated three times. Then the mice were perfused with cold DPBS. The tracheae, lungs and airway lymph nodes were dissected carefully. For single cell suspension preparation, the tissues were cut into small pieces. Lungs were digested using a solution of collagenase II (1 mg/ml, Gibco) and DNase I (1 µl/ml, Invitrogen) for 1 h at 37°C while tracheas were digested with digestion solution composed of papain (20 U/ml, Sigma-Aldrich), EDTA (1.6 mM, Grüssing), L-Cysteine (25 mM, Sigma-Aldrich) and DNase I (0.5 µl/ml, Invitrogen) for 30 min. Airway lymph nodes were solely mechanically disrupted. Subsequently, all tissues except for the blood were filtered through a 70 µm cell strainer to allow for separation into single cells (Falcon, BD Biosciences, Heidelberg, Germany). Red blood cells (RBCs) were removed using RBCs ACK lysis buffer (Thermo Fisher Scientific). Cell counts and viability were estimated using an automatic cell counter (NucleoCounter® NC-200™, Chemomatec, Kaiserslautern, Germany) using the software NucleoView NC-200 version 1.3.0.0 (Chemomatec). Viability of cells isolated from CNO treated mice were stained via a Zombie Aqua Fixable Viabilty Kit (1:100, Biolegend, San Diego, California, USA) according to the manufacturers' protocol. The cells were then fixed using 1% PFA (2% for samples from CNO treated animals).

Single cell suspensions were Fc blocked using CD16/CD32 antibodies for 30-45 min. Subsequently staining of extracellular markers was performed using cocktails of fluorophore-conjugated antibodies for 45 min at 4 °C. For the detection of CD11b+ DCs, neutrophils, monocytes, alveolar and interstitial macrophages the antibodies F4/80-PECy7, CD45-PerCP-Cy5.5, CD11b-FITC, CD86-PE, CD11c-APC-Cy7 and LY6G-APC were used. CCR7+ DCs were labelled with CD11b-Pacific Blue, CD11c-APC-Cy7, CD45-PerCP-Cy5.5, MHCII-PE-Cy7, LY6C-APC, CCR7-PE and F4/80-FITC. CD3-eFluor450, CD4-FITC, TCRβ-APC and TCRγ/δ-FITC were used to identify γδT cells. For T_H17 cells antibodies CD4-FITC and CCR6-APC were applied. ILC3 were detected with CD45-APC-Cy7, CD127-PerCP-Cy5.5, KLRG-BV421 and Lineage-Biotin. Additionally, ILC3s were incubated with Streptavidin-FITC (1:200, BioLegend) for 20 min at 4 °C after extracellular staining. For intracellular staining of

T_H17 cells and ILC3 a transcription factor buffer set (BD Biosciences) in combination with IL-17A-PE-Cy7 for T_H17 cells and T-Bet-APC and RORγT−PE for ILC3 was used according to the manufacturer's protocol. Further informations regarding the antibodies are provided in table 1. The data were collected using a BD FACSverse machine (BD Biosciences) and analyzed using BD FACSuite™ Software version 1.0.6.5230 (BD Biosciences). Data analysis was performed using FlowJo v10.9.0 (FlowJo, LLC, BD Biosciences). The gating strategies can be found in Supplementary Fig. 8-12. Antibody details can be found in Supplementary table 1.

Cytokine measurements

Plasma cytokine IL-17A was measured using luminex discovery assay (mouse premixed multi analyte kit, R&D systems, Minneapolis, MN, USA) according to the manufacturer's protocol. Briefly, all plasma samples were diluted 1:2. 50 µl of standards, controls or diluted samples were added to each well then 50 µl of microparticle cocktail were added to all wells of the microplate and incubated for 2h at room temperature with shaking. After washing, 50 µl of biotin antibody cocktail were added to each well and incubated for 2h at room temperature on a shaker followed by washing. 50 µl of streptavidin-PE were then added and incubated for 30 min at room temperature with shaking followed by washing. The microparticles were suspended by adding 100 µL of wash buffer to each well and incubated for 2 min on the shaker before data acquisition. The measurement was performed using a Magpix machine (Luminex corporation, Austin, TX, USA) and the Milliplex Analyst version 3.5 (Merck Millipore) software.

Isolation of mouse lung dendritic cells and tracheal epithelium

Trpm5$^{+/+}$, *Trpm5*-DREADD and *Panx1*$^{-/-}$ mice were euthanized by an overdose of ketamine/xylazine. Lungs of *Trpm5*$^{+/+}$ and *Panx1*$^{-/-}$ mice and tracheas from *Trpm5*-DREADD mice were collected and processed to obtain single cell suspensions as described above for the

FACS analyses. Lung cells were stained using the following fluorophore conjugated antibodies: F4/80-FITC (1:66) (BioLegend, San Diego, California, USA, RRID: AB_ 893500), CD45-PerCP-Cy5.5 (1:133) (eBioscience, RRID: AB_1107002), CD11b-FITC (1:40) (eBioscience, RRID: AB_464935), CD11b-Pacific blue (1:50) (BioLegend, RRID: AB_755985), CD11c-APC-Cy7 (1:40) (BD Biosciences RRID: AB_10611727) and LY6G-APC (1:40) (eBioscience, RRID: AB_2573307) for 45 min at 4°C.

The gating strategy for isolating pulmonary DCs was: $CD45^+$ $LY6G^-$ $F4/80^-$ $CD11b^+$ $CD11c^+$. Tracheal cells were stained using the following antibodies: CD45-PerCP-Cy5.5 (eBioscience, RRID: AB_1107002), Ep-CAM-PE-Cy7 (BioLegend, RRID: AB_1236477). The Ep-CAM^+ $CD45^-$ cells were collected. FACS was performed on a SH800S cell sorter (Sony biotechnology, San Jose, CA, USA) or on FACS Aria III (BD Biosciences).

Isolated cells were cultured in complete DC media composed of IMDM and glutamax™ (Gibco) with 10% FBS, 50 ng/ml GM-CSF (ImmunoTools, Friesoythe, Germany), 1% penicillin/streptomycin, 1% L-glutamine (Sigma-Aldrich) and 2-mercaptoethanol (Gibco). About $1\text{-}3*10^3$ pulmonary DCs were either co-cultured with $1\text{-}3*10^4$ isolated tracheal epithelium or cultured alone for control experiments. For stimulating tracheal TCs isolated from *Trpm5*-DREADD mice we used CNO (HelloBio) at a concentration of 100 µM for 4 h or for 24 h. Apyrase (Sigma-Aldrich) was used at a concentration of 5 U/ml along with CNO to test the effect of ATP hydrolysis on DCs. To test the effects originating from ACh we used the nicotinic receptor antagonist mecamylamine (Sigma-Aldrich, 10 µM) and the muscarinic receptor antagonist atropine (Sigma-Aldrich, 1 µM). To avoid any direct effect from atropine on DREADD we first stimulated the *Trpm5*-DREADD TC with CNO for five min. Then the tracheal epithelial cells stimulated with CNO were transferred to a well containing sorted pulmonary DCs and supplemented with mecamylamine and atropine in the forementioned concentrations. Then the cells were stained with MHCII-PE-CY7 (BioLegend, RRID: AB_2290801) and CD86 PE (BioLegend, RRID: AB_313151) or CD86-pacific blue

(BioLegend, RRID: AB_493467) or CCR7 PE (BioLegend, RRID: AB_389357). For information about the antibody dilutions see also Table 1. The data collection was performed using a BD FACSverse machine and BD FACSuite™ Software v1.0.6.5230 (BD Biosciences). Data analysis was performed using Flowjo v10.6.2.

Phagocytosis Assay

Bone marrow derived dendritic cells (BM-DCs) were isolated from *Trpm5*$^{+/+}$ mice. Briefly, mice were euthanized by an overdose of isoflurane (Piramal Critical Care Deutschland GmbH) followed by cervical dislocation. The whole bone marrow was flushed out of the marrow cavity from femurs and tibias before being centrifuged at 1200 rpm for 5 min. Precipitated bone marrow derived cells were cultured for 11 days in complete DC medium as described above. For the phagocytosis assay, $5*10^5$ BM-DCs per well were plated in 24 well-plates (Sarstedt, Nümbrecht, Germany) and infected with the *P. aeruginosa* strain NH57388A at a multiplicity of infection (MOI) of 1:20 (20 bacterial cells per one BM-DC) for 1 h at 37°C. Directly before infection, BM-DCs were treated with supernatants obtained from 1 mM denatonium-stimulated explanted tracheas for 30 min at 37°C. Where indicated, tracheal supernatants obtained from 1 mM denatonium-stimulated tracheas was supplemented with apyrase (5 U/ml) (Sigma-Aldrich). The plate was washed twice with DPBS (gibco) and the cells were supplemented with fresh medium containing 100 µg/ml gentamycin (Merck, Darmstadt, Germany). After 30 min of incubation at 37°C, cells were washed with DPBS (gibco) and the phagocytosed bacteria were harvested by lysing the BM-DCs with 0.1% Triton X-100 (Carl Roth, Karlsruhe, Germany). Calculation of the CFU counts was performed by plating serial dilutions of bacteria on agar plates supplemented with 5% sheep blood (BD, Heidelberg, Germany) and overnight incubation at 37°C.

Quantitative RT-PCR

Total RNA was isolated from tracheae and lungs of mice using the RNeasy Micro Kit (Qiagen, Hilden, Germany, REF: 74004) according to the manufacturer's instructions. The synthesis of cDNA essentially carried out as has been reported previously (Hollenhorst et al., 2023)[71] using the SuperScript II reverse transcription kit (Thermo Scientific, REF: 18064014). Briefly, 600 ng of total RNA were transcribed for each experiment. Control reactions were carried out by replacing the reverse transcriptase with nuclease free water.

Gene expression was quantified employing the SYBR®Green two primer-based detection method using the following primer pairs for *Ccl21b*: forward primer 5´-AGGCAAAGAGGGAGCTAGAA-3´ and reverse primer 5´-ATGGCCGTGCAGATGTAATG-3´; and for *Ccl19*: forward 5´-GGTGCTAATGATGCGGAAGA-3´ and reverse 5´-CCCTGCAGCCATCTTCATTA-3´. Normalization was based on the house-keeping gene *B2m* (forward primer 5´-ATTCACCCCCACTGAGACTG-3´and reverse primer 5´-GCTATTTCTTTCTGCGTGCAT-3´). All primers were purchased from IDT (Coralville, IO, USA). Each reaction was set up as follows: 10 µl iTaq Universal SYBR®Green Supermix (Bio-Rad, Laboratories, Inc., Hercules, CA, USA), 6.6 µl nuclease-free water, 2.4 µl primer mix and 1 µl cDNA (from the previous step, see above) (or H_2O) to total of 20 µl. The primer mix for each gene had a concentration of 10 mM for each primer. Reaction conditions included initial denaturation at 95 °C for 30 s followed by 40 cycles at 95 °C for 5 s and 60 °C for 30 s, respectively. All reactions were carried out using the Bio-Rad CFX Connect™ RealTime System (Bio-Rad) and data were collected with the CFX Manager version 3.1 (Bio-Rad).The relative expression was calculated using ΔΔCT method ($\Delta CT = CT_{\text{(gene of interest}} - CT_{\text{(reference gene}}$).

Migration Assay

BM-DCs were isolated and proliferated as described above. In these experiments, $5*10^5$ BM-DCs were plated in 12 well plates (Sarstedt) and incubated overnight at 37°C and 5% CO_2. A small linear scratch (565 ± 12 µM) was performed using 1000 µl sterile pipette tips and the plate was washed with DPBS (gibco) before being supplemented with complete DC medium as described above. Supernatants were collected from tracheae explanted from the following mouse strains: *Trpm5*$^{+/+}$, *Trpm5*$^{-/-}$, *Trpm5*-DREADD and *Trpm5*-DTA. Briefly, tracheae were freshly isolated from euthanized mice and incubated for 10 minutes at 37 °C either in PTP (control) or PTP supplemented either with 1 mM denatonium (for *Trpm5*$^{+/+}$, *Trpm5*$^{-/-}$ and *Trpm5*-DTA mice) and with100 µM CNO (for *Trpm5*-DREADD and *Trpm5*$^{+/+}$ mice). Tracheal supernatants were applied directly to the BM-DCs. The effect of ATP on the migration capacity of BM-DCs was assessed by incubating tracheae from *Trpm5*$^{+/+}$ and *Trpm5*-DTA mice either with 1 mM denatonium or with100 µM CNO in the presence of 5 U/ml apyrase, respectively. Photographs of the linear scratches were taken immediately after applying the tracheal supernatants (representing time point zero, T0) using a DMI-4000B microscope (Leica Microsystems, Wetzlar, Germany) with the software LAS v3.7.0 (Leica Application Suite). The plate was incubated at 37 °C and 5% CO_2 for 24 h (T24). The same scratches were then photographed again. Images were analyzed using the ImageJ software 1.52a (NIH, Bethesda, MD, USA) and the relative wound closure was measured as follow: wound closure % = [(scratch thickness at T0 - scratch thickness at T24) /scratch thickness at T0)] X 100. To estimate the proliferation of the plated dendritic cells, the plated cells were first washed with DPBS (gibco) and then dissociated with 0.5% trypsin (gibco). Cell counts and the percentages for their viability were then determined using NucleoCounter (Chemomatec).

Supplementary table 1: Antibodies used for experiments in this study

Antibodies	Dilution	Clone name	Supplier/Source	RRID or Cat.No.
CD16/32 unconjugated	1:100	93	eBioscience	AB_467133
F4/80-PE-Cy7	1:40	BM8	eBioscience, Thermo Fisher Scientific	AB_469653
CD45-PerCP-Cy5.5	1:133	30-F11	eBioscience	AB_1107002
CD11b-FITC	1:40	M1/70	eBioscience	AB_464935
CD86-PE	1:133	GL1	eBioscience	AB_465768
CD86-Pacific blue	1:50	GL-1	BioLegend	AB_493466
CD11c-APC-Cy7	1:40	HL3	BD Bioscience	AB_10611727
LY6G-APC	1:40	1A8-Ly6g	eBioscience	AB_2573307
CD11b-Pacific Blue	1:50	M1/70	BioLegend	AB_755985
LY6C-APC	1:40	HK1.4	BioLegend	AB_1732087
F4/80-FITC	1:66	BM8	BioLegend	AB_893502
CCR7-PE	1:20	4B12	BioLegend	AB_389357
MHCII-PE-Cy7	1:200	N5/114.15.2	BioLegend	AB_2069376
CD3-eFluor450	1:40	17A2	eBioscience	AB_1272193
CD4-FITC	1:80	RM4-5	eBioscience	AB_464897
TCRβ-APC	1:80	H57-597	BioLegend	AB_313434
TCRγ/δ-FITC	1:50	GL3	BioLegend	AB_313829
CCR6-APC	1:40	29-2L17	BioLegend	AB_1877147
IL17A-PE-Cy7	1:40	eBio17B7	eBioscience	AB_10732356
KLRG-BV421	1:20	2F1/KLRG1	BioLegend	AB_2565613
CD45-APC-Cy7	1:40	30-F11	BioLegend	AB_312981
CD127-PerCP-Cy5.5	1:40	SB/199	BioLegend	AB_1134206

Lineage (CD5, CD3, GR-1, CD45R) Biotin	1:200	CD5: 53-7.3<br>CD3: 145-2C11<br>GR-1: RB6-8C5<br>CD45R: RA3-6B2	BioLegend	AB_312733<br>AB_312669<br>AB_313369<br>AB_312989
T-bet-APC	1:40	4B10	BioLegend	AB_10901173
RORγT–PE	1:50	Q31-378	BD Bioscience	AB_11153137
Ep-CAM-PE-Cy7	1:50	G8.8	BioLegend	AB_1236477
Pannexin 1	1:200	polyclonal	Alomone labs	AB_2340917
Trpm5	1:800	polyclonal	Created in-house	N/A
DCAMKL1	1:1600	polyclonal	abcam	AB_873537
ChAT	1:300 /1:400	polyclonal	Sigma-Aldrich	AB_2079751
CD11c	1:800	N418	eBioscience	AB_467115
ChAT	1:500	13H9L16	ThermoFisher	AB_2848239
Gnat3	1:400	polyclonal	Covalab	pab73402
GFP	1:200	polyclonal	Novusbio	AB_10001164
Anti-rabbit-HRP	1:10000	polyclonal	Sigma-Aldrich	AB_390191
Donkey anti-goat-AF647	1:400	polyclonal	Millipore	AP180SA6
Donkey anti-chicken-FITC	1:200	polyclonal	Jackson ImmunoResearch	AB_2340357
Donkey anti-rabbit-Cy2	1:100	polyclonal	Jackson	711-255-152
Goat anti-armenian-hamster-Cy3	1:800	polyclonal	Jackson	AB_2338989
Donkey anti-goat-Cy3	1:250	polyclonal	Merck	AB_92570
Donkey anti-rabbit-Cy3	1:1000	polyclonal	Merck Millipore	AB_92588

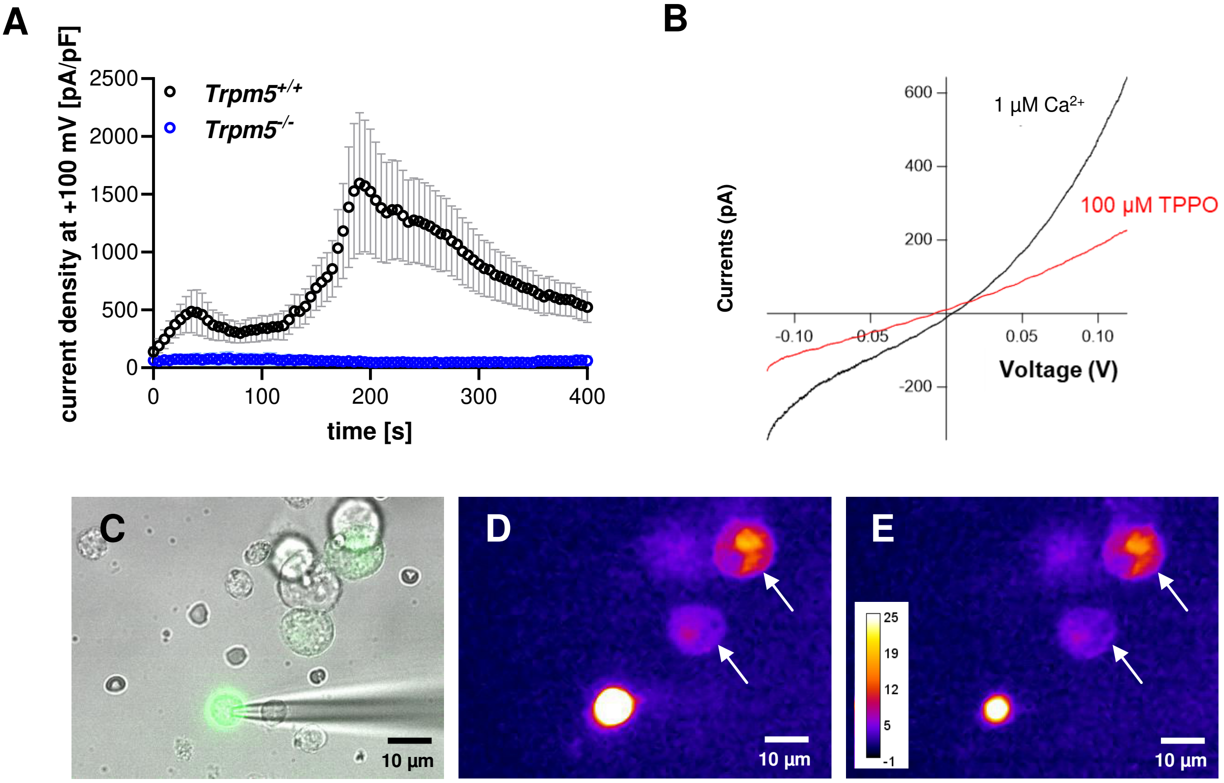

Fig. S1: (**A**) Trpm5 current density of *Trpm5*$^{+/+}$ (black) or *Trpm5*$^{-/-}$ (blue) tuft cells at 100 mV over time after breaking the cell in the whole cell mode. Data are depicted as mean ± SEM. (*Trpm5*$^{+/+}$: n=11 *Trpm5*$^{-/-}$: n=3) (**B**) A representative IV curve showing Trpm5 currents in a tuft cell induced by 1 µM Ca^{2+} (black curve) and in the presence of the Trpm5-specific antagonist TPPO (red curve). (**C**) The bright field image shows a patched tuft cell from a *Trpm5*$^{-/-}$*-ChAT-eGFP* mouse and ATP sensor cells (arrows). Green cells = GFP-expressing tuft cell. (**D-E**) Representative images depicting membrane fluorescence of two ATP sensor cells before (D) and after (E) stimulation of a tuft cell via the application of Ca^{2+} through a patch pipette. No changes in fluorescence were observed. Arrows indicate the cell membranes of the ATP sensor cells at the same time point as shown in Fig. 1F. Source data are provided in the Source Data file.

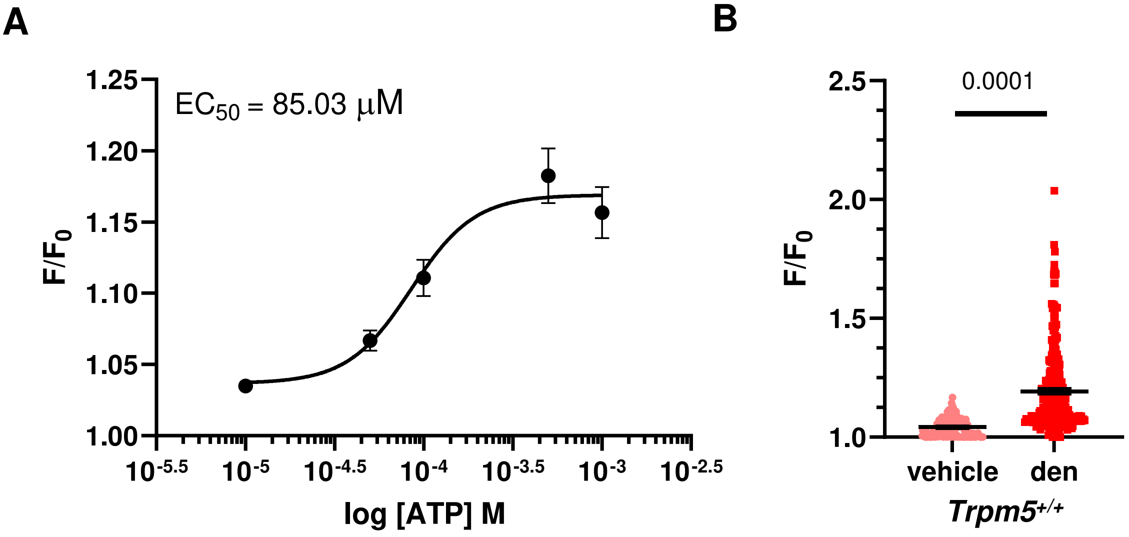

Fig. S2: (**A**) Dose-response curve of ATP sensor cells (HEK293 cells transfected with the ATP sensor iATPSnFR). The cells showed an EC_{50} of 85.03 µM when stimulated with a defined ATP concentration.
(**B**) Supernatants of tracheas treated with 1 mM denatonium (den, *Trpm5*$^{+/+}$: n = 290/3 experiments/3 mice) or vehicle (*Trpm5*$^{+/+}$: n = 169/3 experiments/3 mice) revealed an increase in fluorescence (F/F_0) in ATP sensor cells transfected with the sensor iATPSnFR1.1 after denatonium treatment. (two-tailed unpaired Student's *t*-test) Source data are provided as a Source Data file.

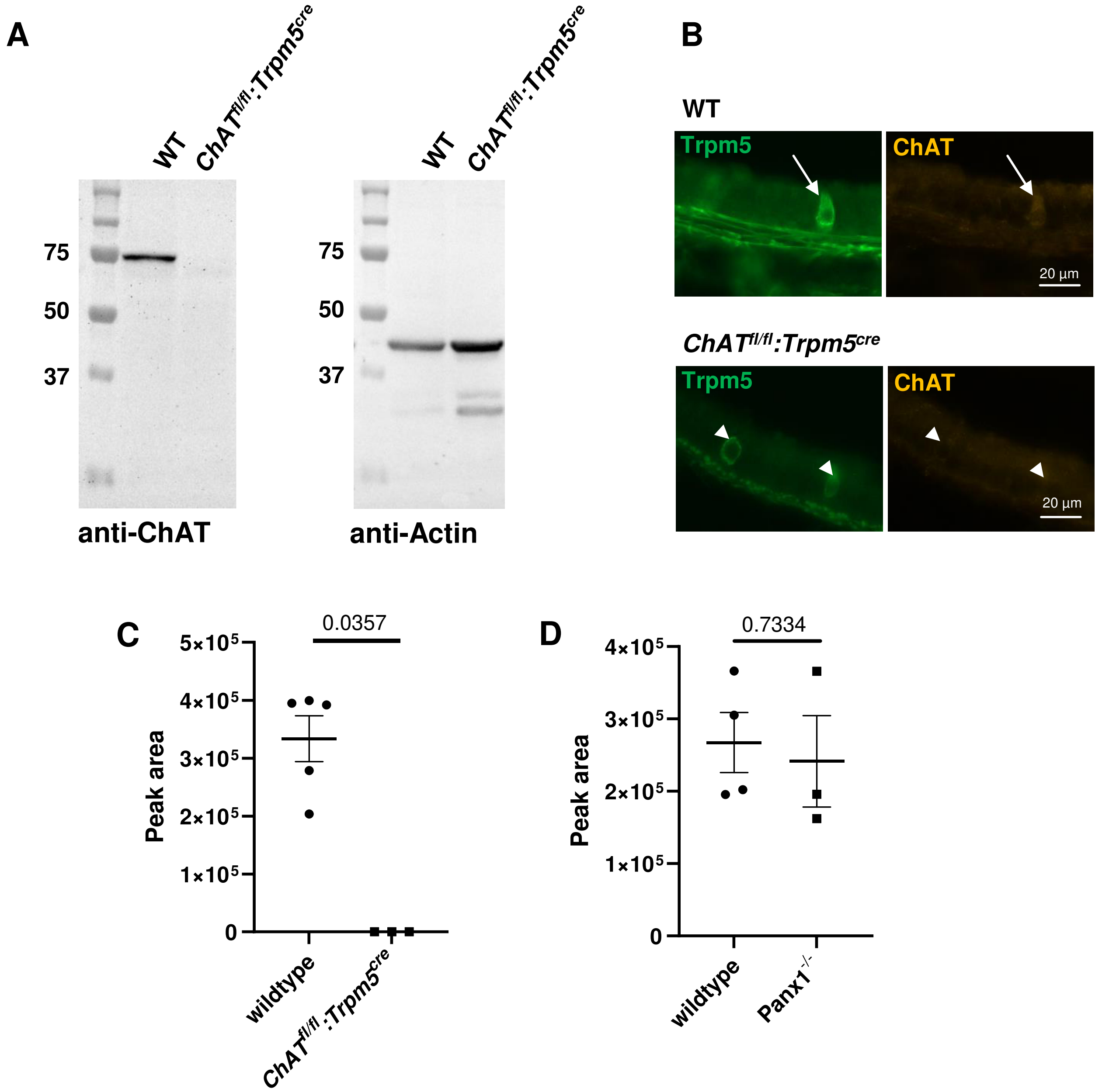

Fig. S3:. Validation of the *ChAT*$^{fl/fl}$:*Trpm5*^{cre} mouse model. (**A**) Immunoblot with homogenates from whole tracheal epithelium from *ChAT*$^{fl/fl}$:*Trpm5*^{cre} and wild-type mice (WT) for ChAT showed ChAT protein in wild-type mice at a size of approximately 72 kD, but not in *ChAT*$^{fl/fl}$:*Trpm5*^{cre} mice. A band for Actin at the expected size of approximately 42 kD was detected in all samples. The experiment was repeated with samples of n=3 mice of each mouse strain. (**B**) Immunochistochemistry for Trpm5 (green) and ChAT (yellow) in wild-type and *ChAT*$^{fl/fl}$:*Trpm5*^{cre} mice showed double-positive cells (arrows) in wild-type, but not in *ChAT*$^{fl/fl}$:*Trpm5*^{cre} mice (n=4). (**C**) ACh is detected after stimulation with denatonium (1 mM) in tracheal supernatants from wild-type (n=5) but not from *ChAT*$^{fl/fl}$:*Trpm5*^{cre} mice (n=3). MALDI-TOF, two-tailed unpaired Student's *t*-test (**D**) ACh levels in tracheal supernatants from *pannexin1*-deficient (*Panx1*$^{-/-}$) mice (n=4) after stimulation with 1 mM denatonium does not differ from those in wild-type mice (n=3). MALDI-TOF, two-tailed unpaired Student's *t*-test, Source data are provided in the Source Data file.

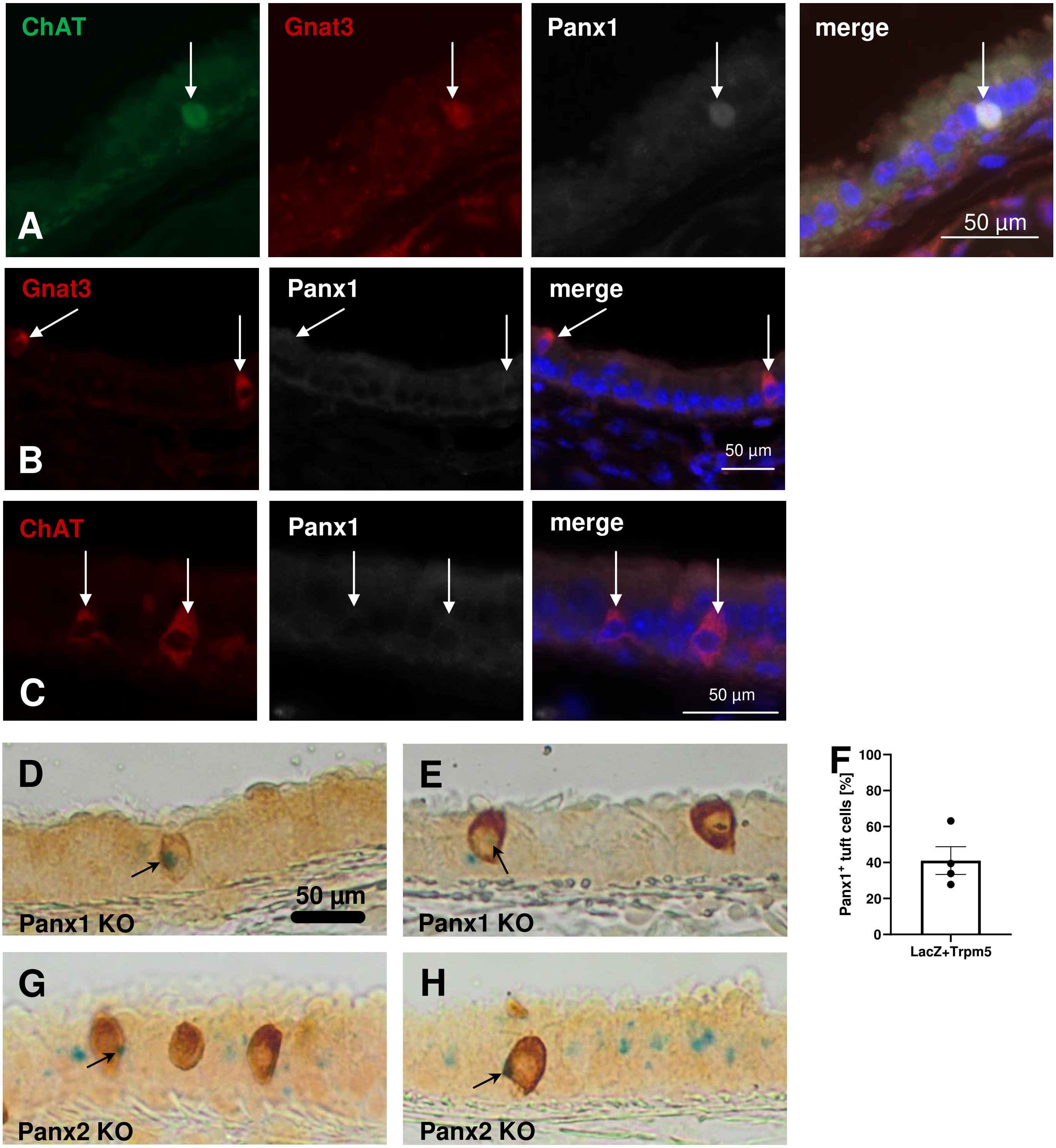

ChAT
Gnat3
Panx1
merge
A
50 µm
Gnat3
Panx1
merge
B
50 µm
ChAT
Panx1
merge
C
50 µm
D
50 µm
Panx1 KO
E
Panx1 KO
F
100
80
60
40
20
0
Panx1+ tuft cells [%]
LacZ+Trpm5
G
Panx2 KO
H
Panx2 KO

Fig. S4: Panx1 immunofluorescence staining and Panx1 or Panx2 LacZ-staining in combination with anti-Trpm5 immunohistochemistry (**A**) Immunofluorescence staining revealed overlay of pannexin (Panx1) staining (white) and tuft cells (ChAT, cholineacetyltransferase, green, Gnat3, red) in a subpopulation of cells (arrows), but also some additional PANX1 staining in ChAT-negative cells (star). (**B-C**) There was no Panx1 staining (white) in tracheal sections of Panx1$^{-/-}$ mice stained for Gnat3 (**A**) or for ChAT (**C**) as tuft cell markers (red, arrows). (**D-E**) LacZ-positive signals indicating *Panx1* promoter activity and, consequently, pannexin-1 gene transcription are present throughout the tracheal epithelium. Some of the lacZ signals are present in tuft cells as assessed using immunohistochemistry to indicate the presence of Trpm5 (black arrows). LacZ-signals, however, are not exclusive to tuft cells but can also be found in the vicinity of Trpm5-positive cells. (**F**) Quantification of double-positive LacZ+/Trpm5+ tuft cells *Panx1*$^{-/-}$ mouse tracheas (n=4). (**G-H**) LacZ-positive signals indicating *Panx2* promoter activity and, consequently, pannexin-2 gene transcription are present throughout the tracheal epithelium. Some of the lacZ signals are present in tuft cells as assessed using immunohistochemistry to indicate the presence of Trpm5 (black arrows) LacZ-signals, however, are not exclusive to tuft cells but can also be found in the vicinity of Trpm5-positive cells. Source data are provided in the Source Data file.

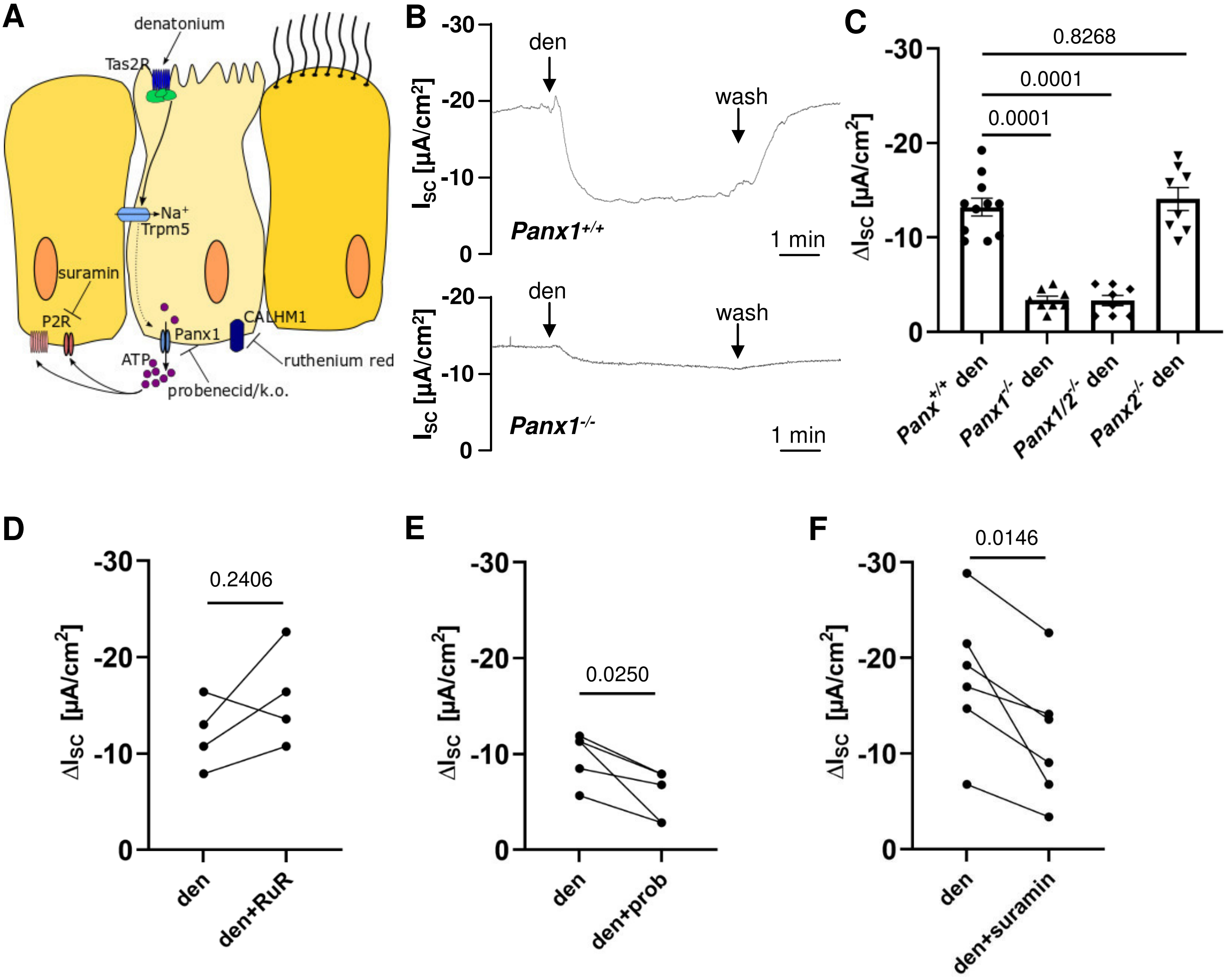

Fig. S5: Tuft cell released ATP modulates transepithelial ion transport. (**A**) Schematic drawing of the mechanism by which denatonium induces ATP release. (**B**) Representative current traces (I_{SC}) of a wild-type (*Panx1*$^{+/+}$) and of a *Panx1*$^{-/-}$ mouse trachea upon apical application of 1 mM denatonium (den). (**C**) In *Panx1*$^{-/-}$ (n=4) and *Panx1*$^{-/-}$*2*$^{-/-}$ mice (n=4) the denatonium-induced current (1 mM, apical, ΔI_{SC}) was reduced compared to wild type controls (*Panx*$^{+/+}$, n=11, while the denatonium-induced effect in *Panx2*$^{-/-}$ mice (n=4) was similar to wild-type mice. (one-way ANOVA) (**D**) The CALHM1 inhibitor Ruthenium Red (RuR, 20 µM, apical and basolateral) did not change the denatonium-induced current compared to control conditions (n=4, two-tailed paired Student's *t*-test). (**E**) Application of the pannexin inhibitor probenecid (200 µM, apical and basolateral) led to a significant reduction of the denatonium-induced current in wildtype mice (n=5, two-tailed paired Student's *t*-test). (**F**) In the presence of the non-selective P2 purinergic receptor antagonist suramin (100 µM, basolateral) the denatonium-induced effect was diminished (n=6, two-tailed paired Student's *t*-test). Source data are provided in the Source Data file.

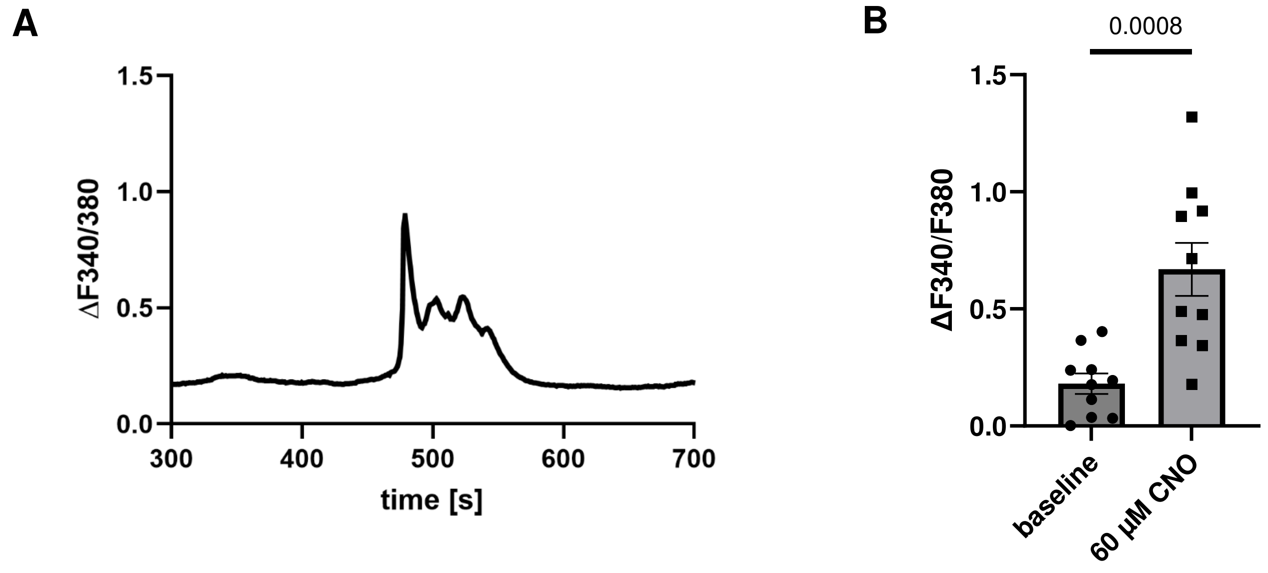

Fig. S6: Ca^{2+}-Imaging of tuft cells from *Trpm5*-DREADD mice stimulated with CNO. (**A**) Representative curve of $[Ca^{2+}]_i$ levels of a tuft cell in response to CNO (60 µM). (**B**) CNO (60 µM) led to a significant, transient increase in $[Ca^{2+}]_i$ levels in tuft cells from *Trpm5*-DREADD-tGFP mice (n=10 cells from 3 mice). (two-tailed, paired Student's *t*-test). Source data are provided in the Source Data file.

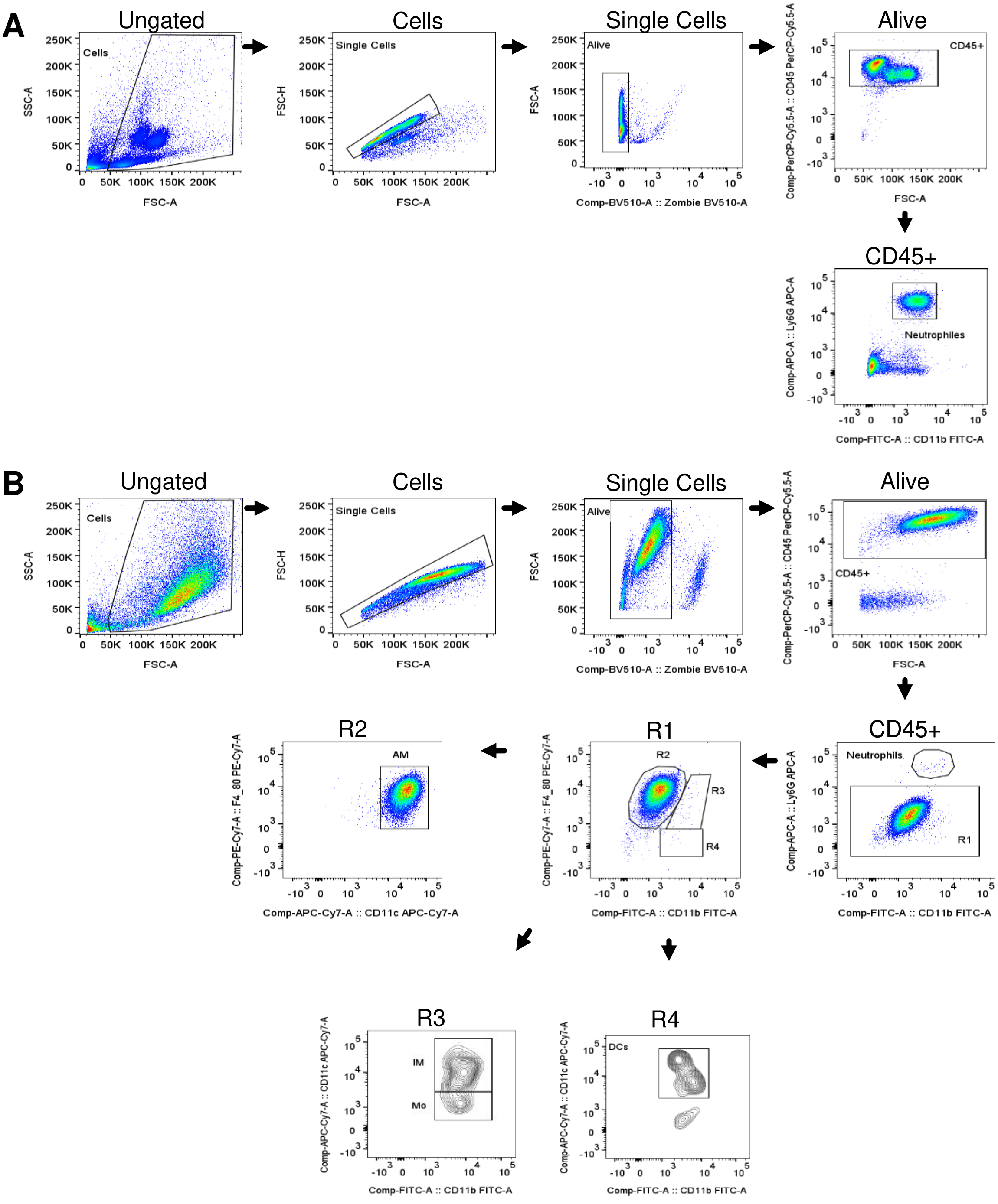

Fig. S7: (**A**) Gating strategy for flow cytomertric identification of blood neutrophils. After exclusion of cell debris, doublets and dead cells, neutrophils were identified as CD45$^+$ CD11b$^+$ Ly6G$^+$. (**B**) Gating strategy for analysis of (CD45$^+$ CD11b$^+$ Ly6G$^+$), alveolar macrophages (AM) (CD45$^+$ CD11b$^-$ Ly6G$^-$ F4/80$^+$ CD11c^{high}), monocytes (CD45$^+$ CD11b$^+$ Ly6G$^-$ F4/80$^+$ CD11c$^-$), interstitial macrophages (IM) (CD45$^+$ CD11b$^+$ Ly6G$^-$ F4/80$^+$ CD11c$^+$) and CD11b+ DCs (CD45$^+$ Ly6G$^-$ CD11b$^+$ F4/80$^-$ CD11c$^+$) in bronchoalveolar lavage fluid (BALF).

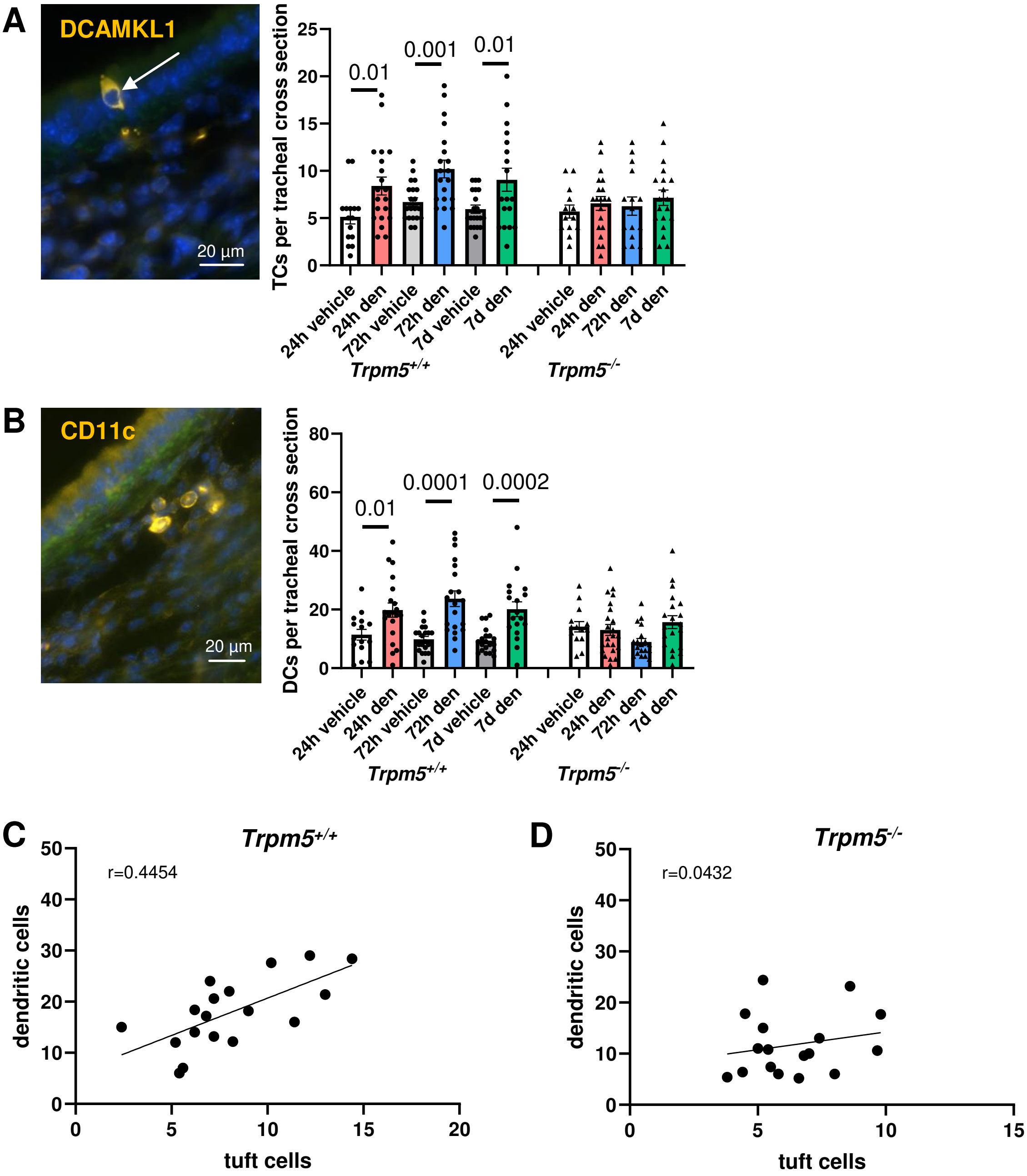

Fig. S8: Dendritic cell numbers correlate with tuft cell numbers. (**A**) Representative immunofluorescence staining for tuft cells (DCAMKL1) in tracheal cross sections and quantification of DCAMKL1$^+$ cells in *Trpm5*$^{+/+}$ and *Trpm5*$^{-/-}$ mice treated with 1 mM denatonium after 24h, 72h, 7d or with vehicle (PBS) after 24h (*Trpm5*$^{+/+}$ and *Trpm5*$^{-/-}$ mice), 72h or 7d (*Trpm5*$^{+/+}$ mice) (*Trpm5*$^{+/+}$ 24h vehicle n=15, 24h den n=20, 72h vehicle n=20, 72h den n=20, 7d vehicle n=20, 7d den n=18, *Trpm5*$^{-/-}$ 24h vehicle n=13, 24h den n=20, 72h den n=15, 7d den n=19 sections of 4 mice per condition). (**B**) Representative immunofluorescence staining of dendritic cells (CD11c) in tracheal cross sections and quantification of CD11c$^+$ cells in *Trpm5*$^{+/+}$ and *Trpm5*$^{-/-}$ mice treated with 1 mM denatonium after 24h, 72, 7d or with vehicle (PBS) after 24h (*Trpm5*$^{+/+}$ and *Trpm5*$^{-/-}$ mice), 72h or 7d (*Trpm5*$^{+/+}$ mice) (*Trpm5*$^{+/+}$ 24h vehicle n=15, 24h den n=20, 72h vehicle n=20, 72h den n=20, 7d vehicle n=20, 7d den n=18, *Trpm5*$^{-/-}$ 24h vehicle n=14, 24h den n=23, 72h den n=19, 7d den n=20 sections of 4 mice per condition). (**C**) Correlation analysis of tuft cell and dendritic cell numbers of *Trpm5*$^{+/+}$ mice revealed a moderate positive linear correlation with Pearson's r=0.4454. (**D**) There was no correlation between tuft cell and dendritic cell numbers in *Trpm5*$^{-/-}$ mice (r=0.0432). A, B: Two-tailed unpaired Student's t-test for *Trpm5*$^{+/+}$ and one-way ANOVA for *Trpm5*$^{-/-}$. Source data are provided in the Source Data file.

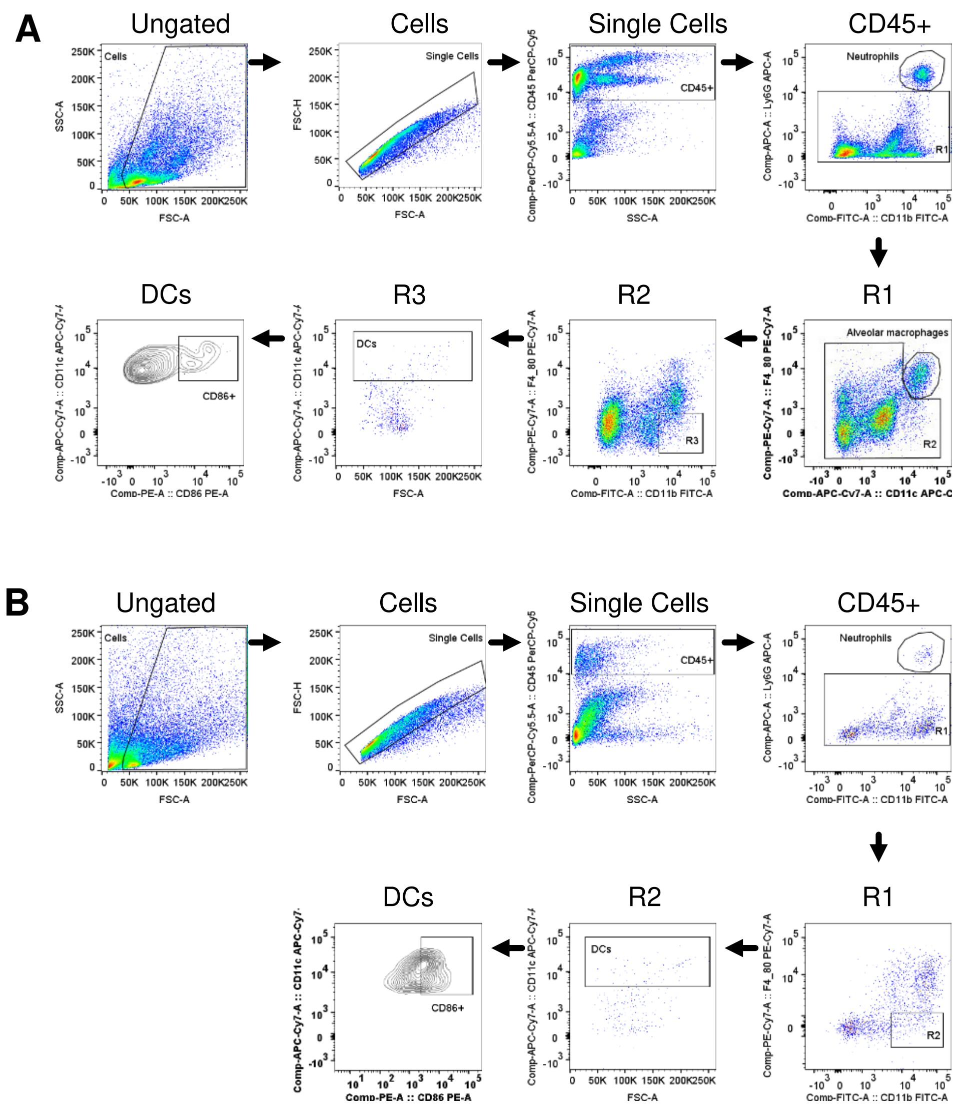

Fig S9: Gating strategy for flow cytomertric identification of CD11b⁺ DCs in mouse lungs (**A**) and tracheae (**B**). After exclusion of cell debris and doublets, neutrophils (CD45⁺ Ly6G⁺ CD11b⁺) and alveolar macrophages (CD45⁺ Ly6G⁻ F4/80⁺ CD11c⁺) in case of lung tissue or only neutrophils in case of tracheal tissue were distinguished and also excluded. Subsequently, CD11b⁺ DCs (CD45⁺ Ly6G⁻ F4/80⁻ CD11b⁺ CD11c⁺) were identified, and the percentage of CD86⁺ DCs was determined.

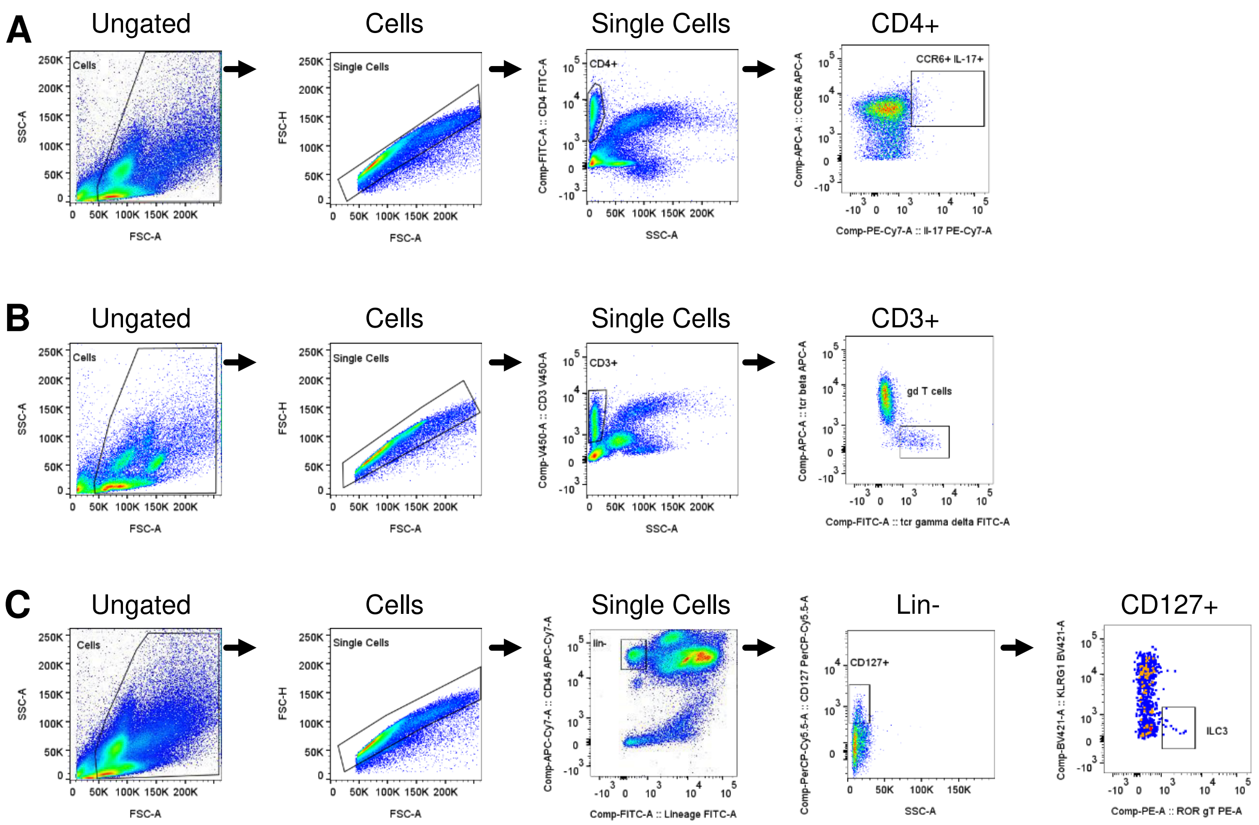

Fig S10: (**A**) Gating strategy for flow cytomertric identification of T_H17 in lung tissue. After exclusion of cell debris and doublets, CD4$^+$ T_H cells were gated and the T_H17 subtype was identified as CCR6$^+$ IL-17$^+$. (**B**) Gating strategy for analysis of γδ T cells within mouse lungs (CD3$^+$ TCR β$^-$ TCR γ/δ$^+$). (**C**) Gating strategy for identification of pulmonary ILC3. CD45$^+$ Lin$^-$ cells were gated and the CD127$^+$ RORg$^+$ T cells were identified as the ILC3 population.

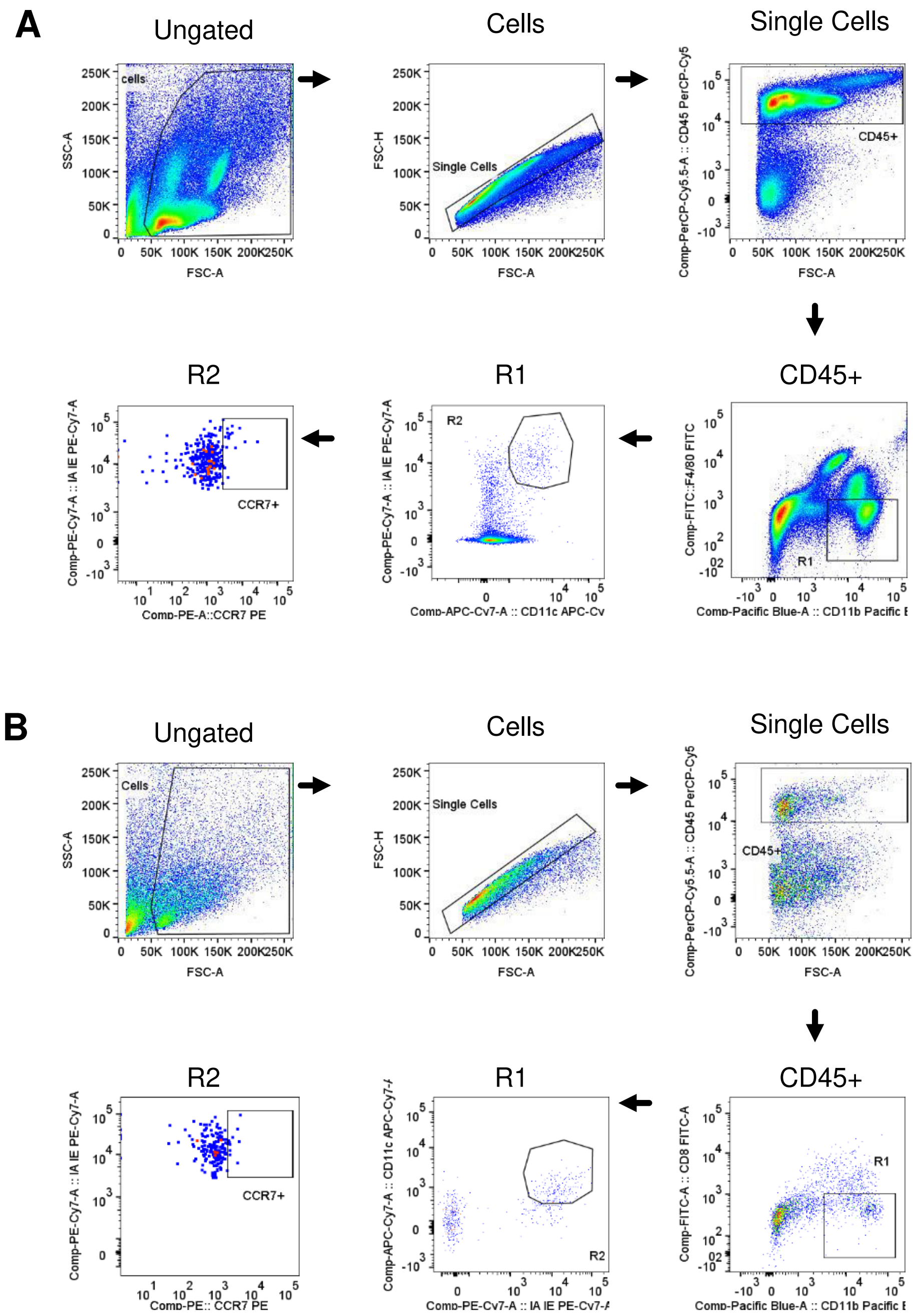

Fig. S11: Gating strategy for flow cytomertric identification of CCR7⁺ DCs in lungs (**A**) and tracheae (**B**). Cell debris and doublets were excluded followed by gating of CD45⁺ immune cells. After that, CD11b⁺ DCs were identified as CD11b⁺ F4/80⁻ CD11c⁺ IA/IE⁺ and then CCR7⁺ cells were gated.

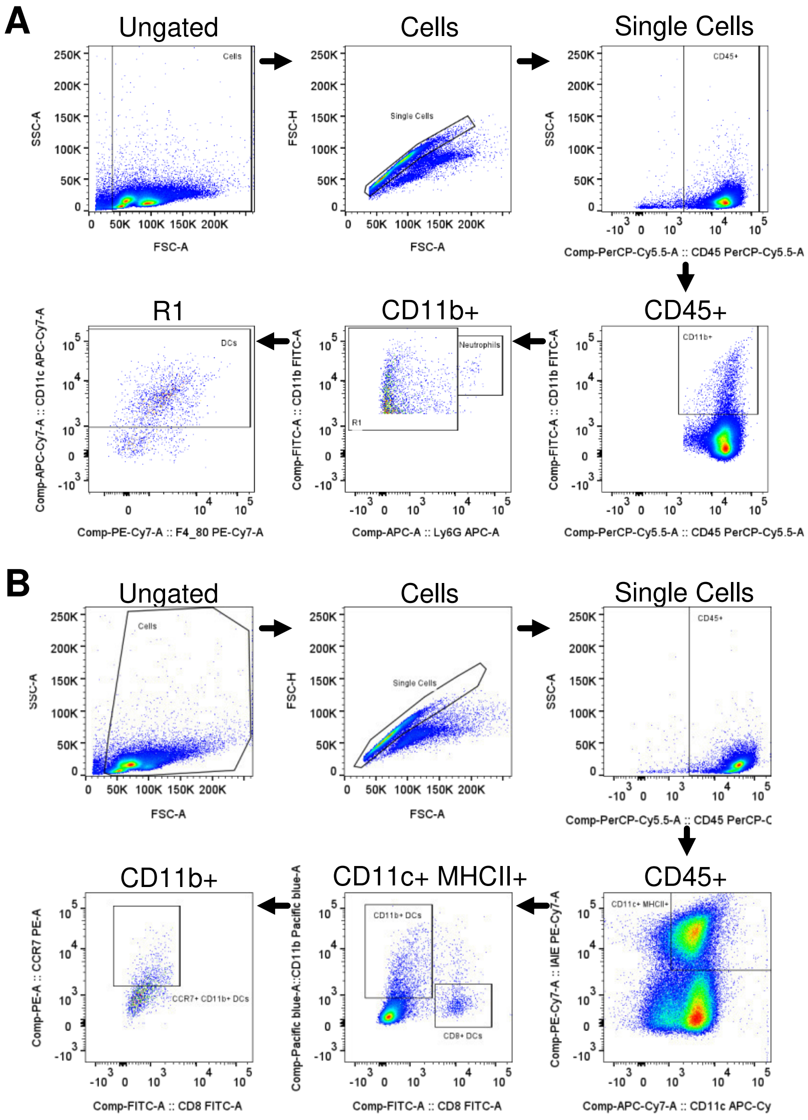

Fig. S12: (**A**) Gating strategy for flow cytomertric identification of $CD11b^+$ DCs in airway draining lymph nodes. After exclusion of cell debris, doublets and neutrophils ($CD45^+$ $CD11b^+$ $Ly6G^+$) $CD11b^+$ DCs ($CD45^+$ $CD11b^+$ $Ly6G^-$ $CD11c^+$) were identified. (**B**) Gating strategy for analyses of $CCR7^+$ DCs within mouse lymph nodes. First, cell debris and doublets were excluded, followed by identification of $CD11b^+$ DCs ($CD45^+$ $CD11c^+$ $MHCII^+$). DCs were then gated for CCR7 expression.

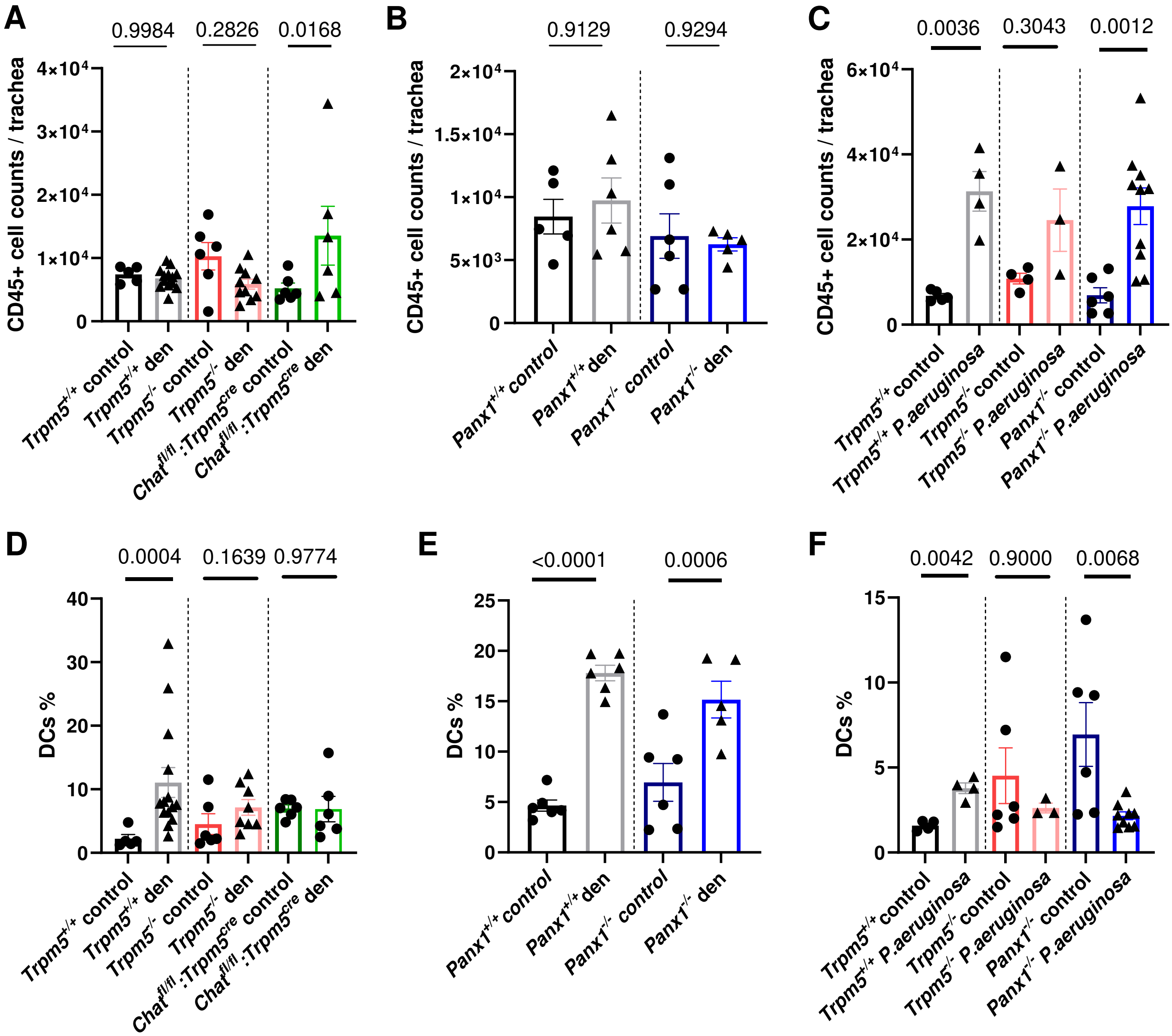

Fig S13: FACS analyses of total leukocytic cell counts (CD45$^+$) and percentages of DCs within mouse tracheae after stimulation of tuft cells by 1 mM denatonium or *Pseudomonas aeruginosa* NH57388A infection. (**A**) Total leukocytic cell counts (CD45$^+$) in tracheae of denatonium-treated (den n=13) or control (n=5) *Trpm5*$^{+/+}$, *Trpm5*$^{-/-}$ (control n=6, den n=10) and *Chat*$^{fl/fl}$:*Trpm5*^{cre} mice (control n=6, den n=6) 72 hours after intratracheal application. One-way ANOVA. (**B**) Total leukocytic counts in tracheae of control (n=6) and denatonium-treated (den n=5) *Panx1*$^{-/-}$ and *Panx1*$^{+/+}$ mice (control n=5, den n=6). One-way ANOVA. (**C**) Total leukocytic counts in *Trpm5*$^{+/+}$ (control n=5, P.a. n=4), *Trpm5*$^{-/-}$ (control n=4, P.a. n=3) and *Panx1*$^{-/-}$ mice (control n=6, P.a. n=10) three days post infection or in healthy controls. One-way ANOVA. (**D**) Percentage of DCs in tracheae of control (n=5) or denatonium-treated (den n=14) *Trpm5*$^{+/+}$, *Trpm5*$^{-/-}$ (control n=6, den n=8) and *Chat*$^{fl/fl}$:*Trpm5*^{cre} mice (control n=6, den n=6). Kruskal-Wallis test. (**E**) Percentage of DCs in tracheae of control (n=6) or denatonium-treated (den n=5) *Panx1*$^{-/-}$ and *Panx1*$^{+/+}$ mice (control n=6, den n=6). One-way ANOVA. (**F**) Percentage of DCs in tracheae of infected or uninfected controls in *Trpm5*$^{+/+}$ (control n=4, P.a. n=4), *Trpm5*$^{-/-}$ (control n=6, P.a. n=3) and *Panx1*$^{-/-}$ mice (control n=6, P.a. n=9). Kruskal-Wallis test. Source data are provided in the Source Data file.

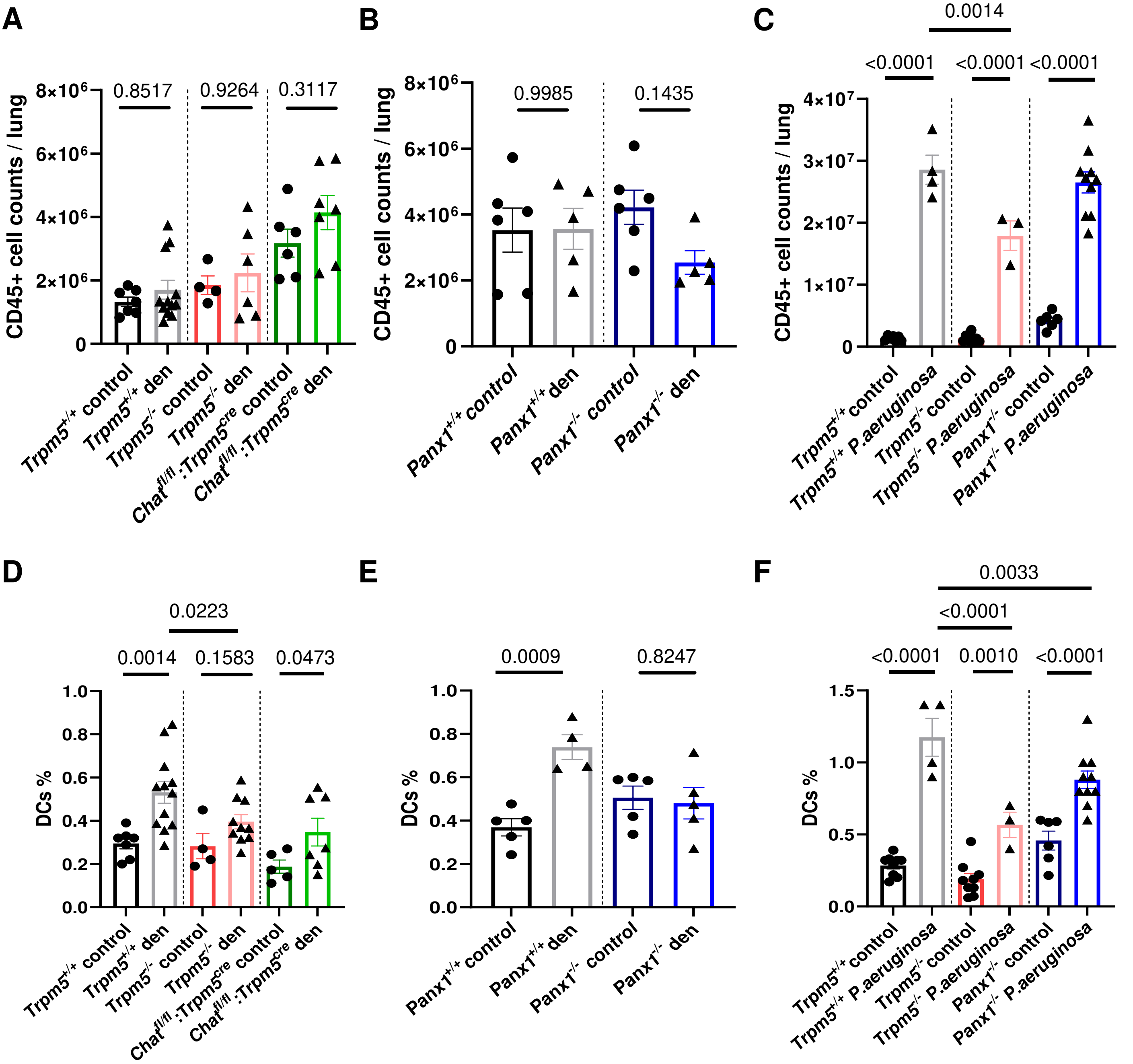

Fig S14: FACS analyses of total leukocytic cell counts ($CD45^{+}$) and percentages of DCs within mouse lungs following intratracheal inhalation of 1mM denatonium or *Pseudomonas aeruginosa* NH57388A infection. (**A**) Total leukocytic cell counts ($CD45^{+}$) in lungs of denatonium-treated (den n=12) or control (n=7) *Trpm5*$^{+/+}$, *Trpm5*$^{-/-}$ (control n=4, den n=6) and *Chat*$^{fl/fl}$*:Trpm5*^{cre} mice (control n=6, den n=7) after three days. One-way ANOVA. (**B**) Total leukocytic counts in lungs of denatonium-treated (n=5) or control (n=6) *Panx1*$^{-/-}$ and *Panx1*$^{+/+}$ mice (control n=6, den n=5). One-way ANOVA. (**C**) Total leukocytic counts three days post infection or in uninfected controls of *Trpm5*$^{+/+}$ (control n=7, P.a. n=4), *Trpm5*$^{-/-}$ (control n=8, P.a. n=3) and *Panx1*$^{-/-}$ mice (control n=6, P.a. n=10). One-way ANOVA. (**D**) Percentage of DCs in lungs of controls (n=7) or denatonium-treated (den n=12) *Trpm5*$^{+/+}$, *Trpm5*$^{-/-}$ (control n=4, den n=10) and *Chat*$^{fl/fl}$*:Trpm5*^{cre} mice (control n=5, den n=7). One-way ANOVA. (**E**) Percentage of DCs in lungs of controls (n=5) or denatonium-treated (n=5) *Panx1*$^{-/-}$ and *Panx1*$^{+/+}$ mice (control n=5, den n=4). One-way ANOVA. (**F**) Percentage of DCs in lungs of infected or uninfected controls in *Trpm5*$^{+/+}$ (control n=9, P.a. n=4), *Trpm5*$^{-/-}$ (control n=9, P.a. n=3) and *Panx1*$^{-/-}$ mice (control n=6, P.a. n=10). One-way ANOVA. Source data are provided in the Source Data file.

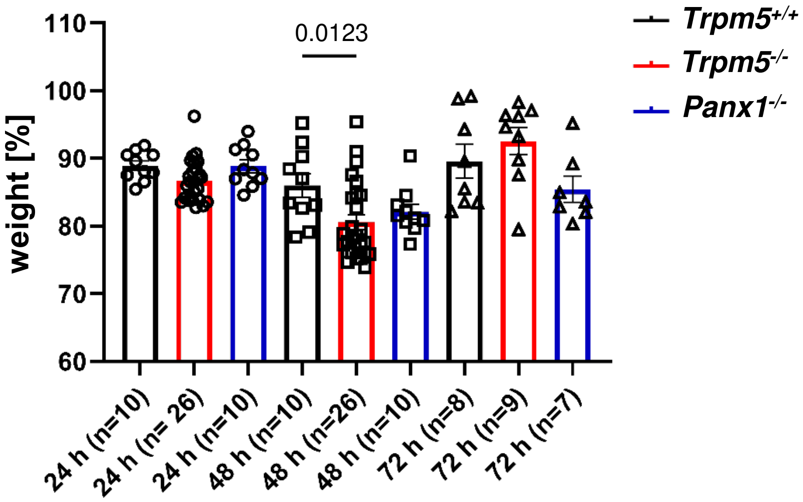

Fig. S15: Weight of infected wild-type (*Trpm5*$^{+/+}$/*Panx1*$^{+/+}$)), Trpm5-deficient (*Trpm5*$^{-/-}$) and pannexin1-deficient (*Panx1*$^{-/-}$) mice (n=7-26). Two-tailed Mann-Whitney test. Source data are provided in the Source Data file.

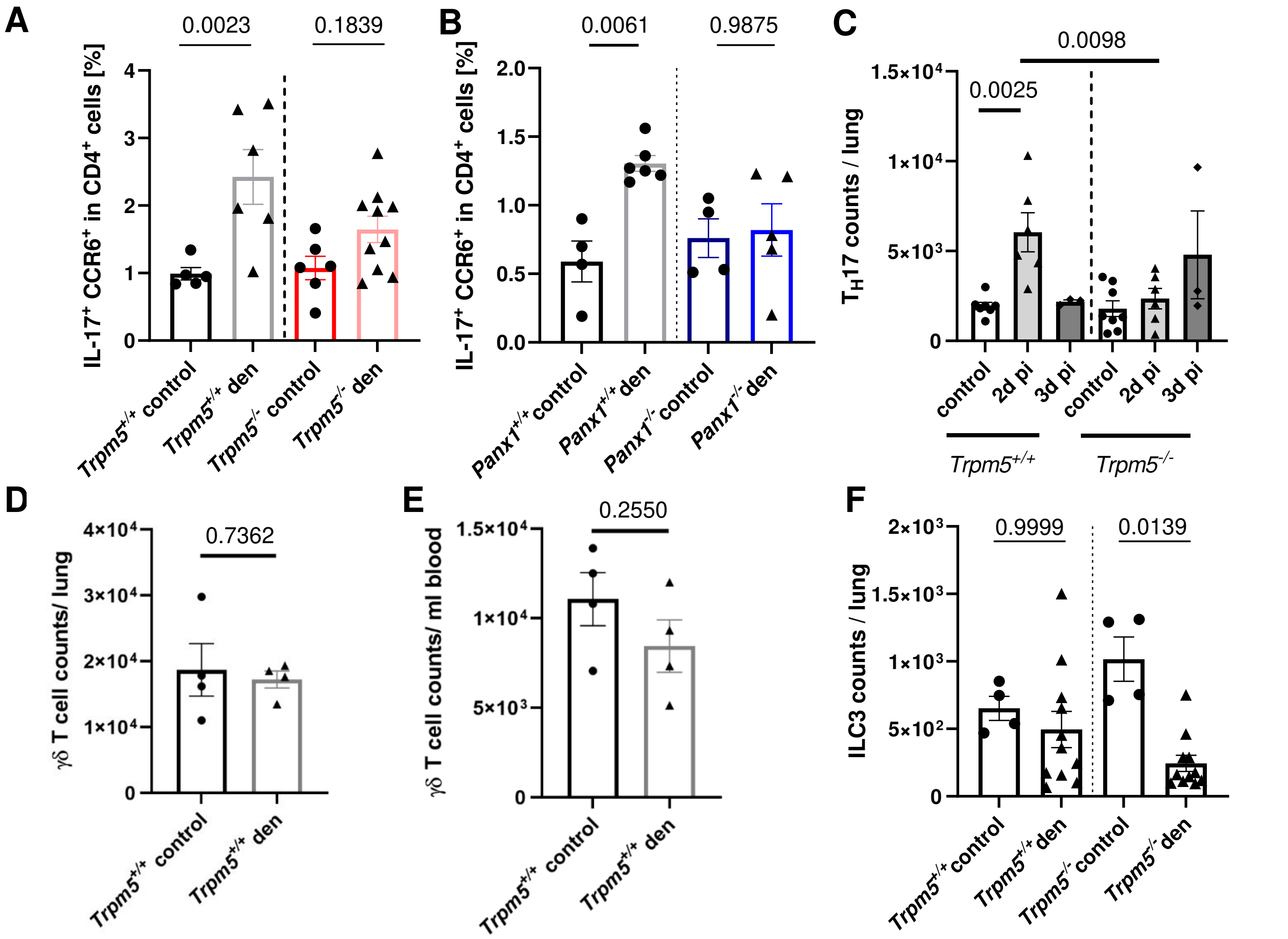

Fig. S16: **(A)** Percentages of T_H17 cells as a fraction of CD4$^+$ cells in lungs of denatonium-treated (den) or control *Trpm5*$^{+/+}$ and *Trpm5*$^{-/-}$ mice (*Trpm5*$^{+/+}$ control n=5, den n=6, *Trpm5*$^{-/-}$ control n=6, den n=10). One-way ANOVA. (**B**) Percentages of T_H17 cells in denatonium-treated or control *Panx1*$^{+/+}$ (control n=4, den n=6) and *Panx1*$^{-/-}$ mice (control n=4, den n=5). One-way ANOVA. (**C**) FACS analyses of T_H17 cell counts in lungs of *Trpm5*$^{+/+}$ and *Trpm5*$^{-/-}$ mice 2 days (2d pi) and 3 days post infection (3d pi) with *P. aeruginosa*, compared to untreated controls showed increased T_H17 cell levels 2d pi in *Trpm5*$^{+/+}$ mice (*Trpm5*$^{+/+}$ control n=7, 2d pi n=6, 3d pi n=3, *Trpm5*$^{-/-}$ control n=8, 2d pi n=6, 3d pi n=3). One-way ANOVA (**D**, **E**) γδ T cell levels in *Trpm5*$^{+/+}$ mice remained unaltered in the lungs and in the blood tree days after treatment with denatonium compared to untreated control animals in FACS analyses (n=4 for control and denatonium-treated animals). Two-tailed unpaired Student's *t*-test. (**F**) FACS analyses of lungs of denatonium-treated *Trpm5*$^{+/+}$ and *Trpm5*$^{-/-}$ mice revealed no changes in ILC3 numbers after denatonium treatment compared to controls (*Trpm5*$^{+/+}$ control n=4, den n=11, *Trpm5*$^{-/-}$ control n=4, den n=11). (Kruskal-Wallis test) Source data are provided in the Source Data file.

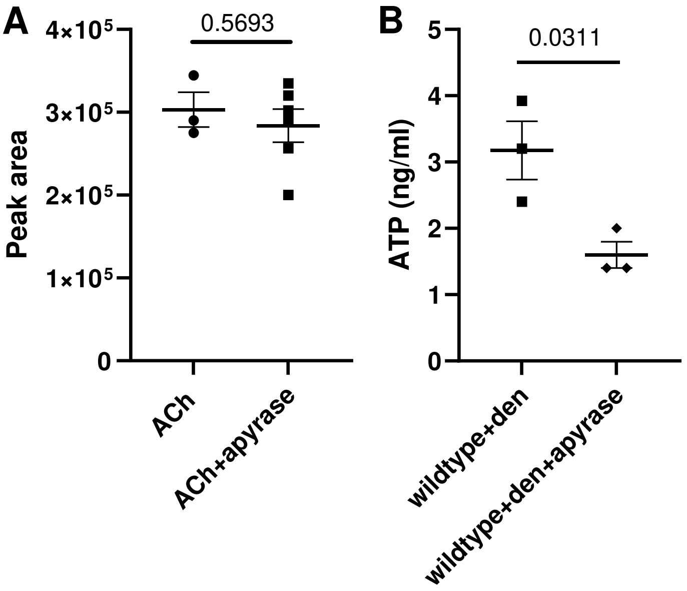

Fig. S17:. (**A**) The ACh concentration (10 nM) remains unaltered in a solution containing apyrase (5 U/ml). ACh (n=3), ACh + apyrase (n=6). MALDI-TOF, two-tailed unpaired Student's *t*-test. (**B**) The ATP concentration in supernatants from denatonium-treated tracheae (n=3) was reduced by apyrase (5 U/ml) (n=3). ELISA, two-tailed unpaired Student's *t*-test. Source data are provided in the Source Data file.

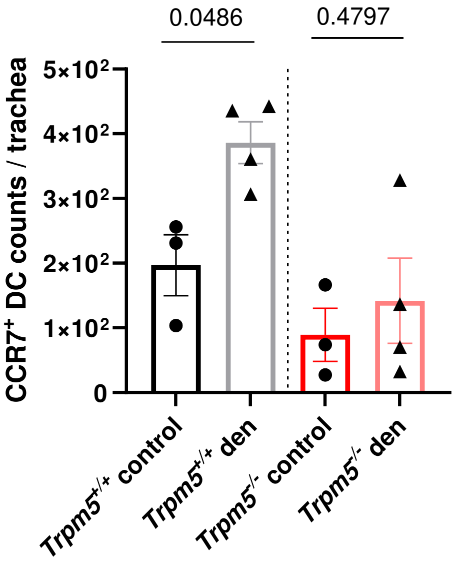

Fig. S18: FACS analyses of $CCR7^+$ DC numbers in tracheae from *Trpm5*$^{+/+}$ and *Trpm5*$^{-/-}$ mice revealed an increase in $CCR7^+$ DCs three days after treatment with denatonium in *Trpm5*$^{+/+}$ but not in *Trpm5*$^{-/-}$ mice. (*Trpm5*$^{+/+}$ ctrl n=3, den n=4, *Trpm5*$^{-/-}$ ctrl n=3, den n=4). One-way ANOVA. Source data are provided in the Source Data file.

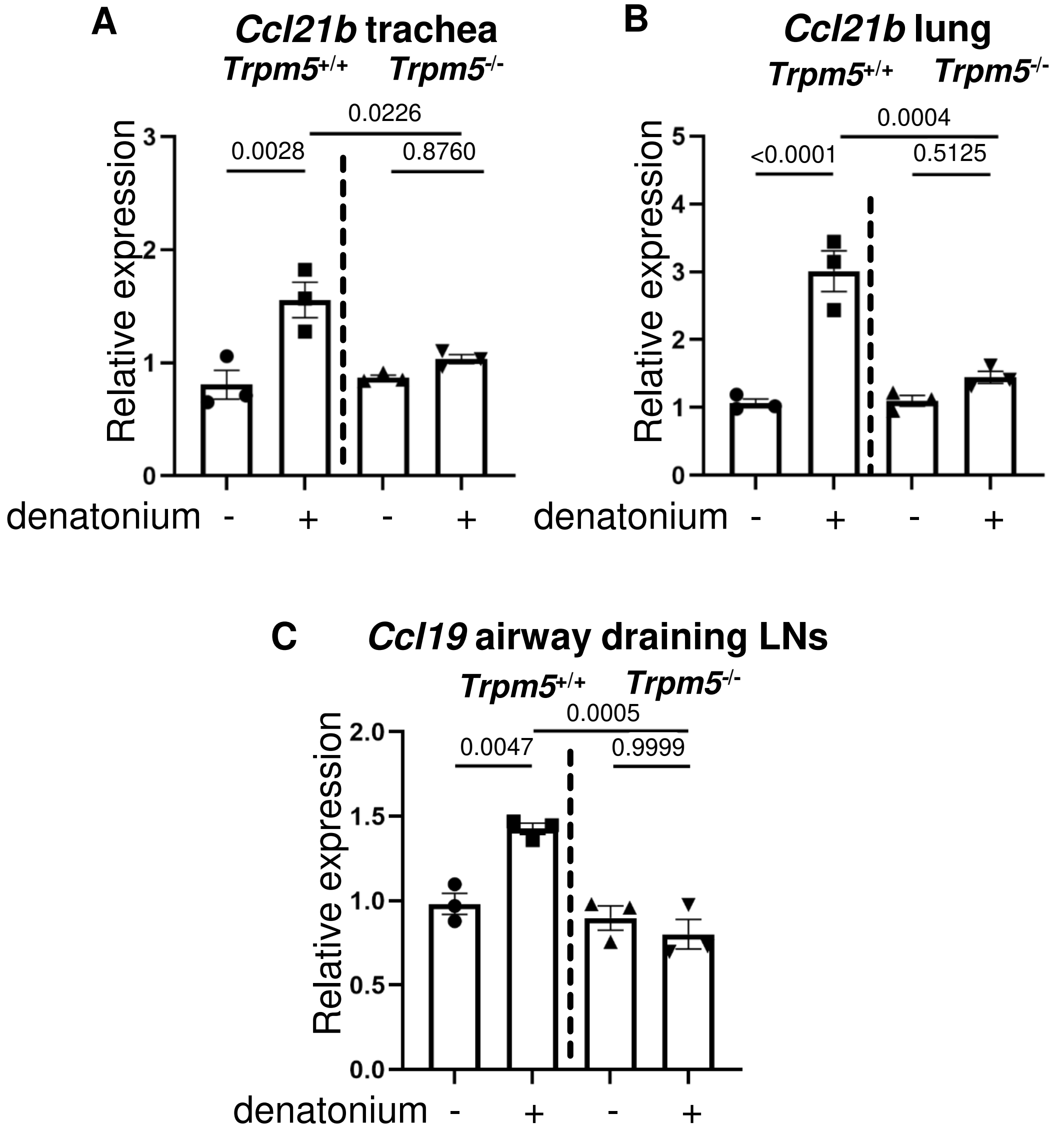

Fig. S19: Relative expression of *Ccl21b* in whole tracheae (**A**) and lungs (**B**) of *Trpm5*$^{+/+}$ and *Trpm5*$^{-/-}$ mice 3 days after the intratracheal application of 1 mM denatonium (+) or control (-) (n=3, each point represent the average of two technical replicates). (**C**) Relative expression of *Ccl19* in airway draining lymph nodes (LN) of *Trpm5*$^{+/+}$ and *Trpm5*$^{-/-}$ mice 3 days after the intratracheal application of 1 mM denatonium (+) or control (-) (n=3, each point represent the average of two technical replicates). One-way ANOVA. Source data are provided in the Source Data file.

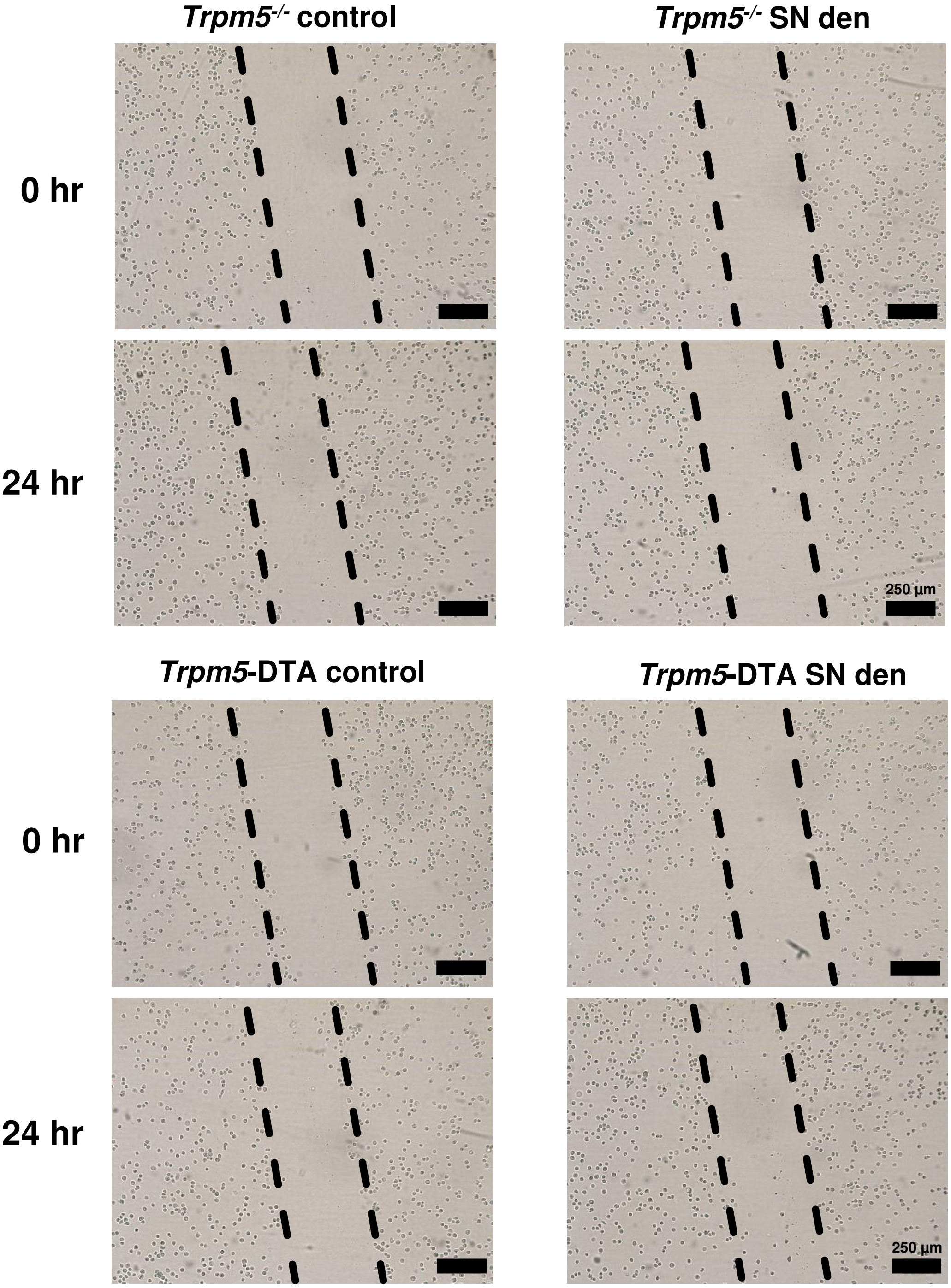

Fig. S20: Migration assay with dendritic cells. Representative images of dendritic cells before (0 h) and after stimulation (24 h) with supernatants (SN) obtained from denatonium- (den, 1 mM) or vehicle-treated (control) tracheae of Trpm5$^{-/-}$ and *Trpm5*-DTA mice. Scale bar = 250 µm. The quantitative analyses of the migration assay can be found in Figure 7e. (n=3 independent experiments)

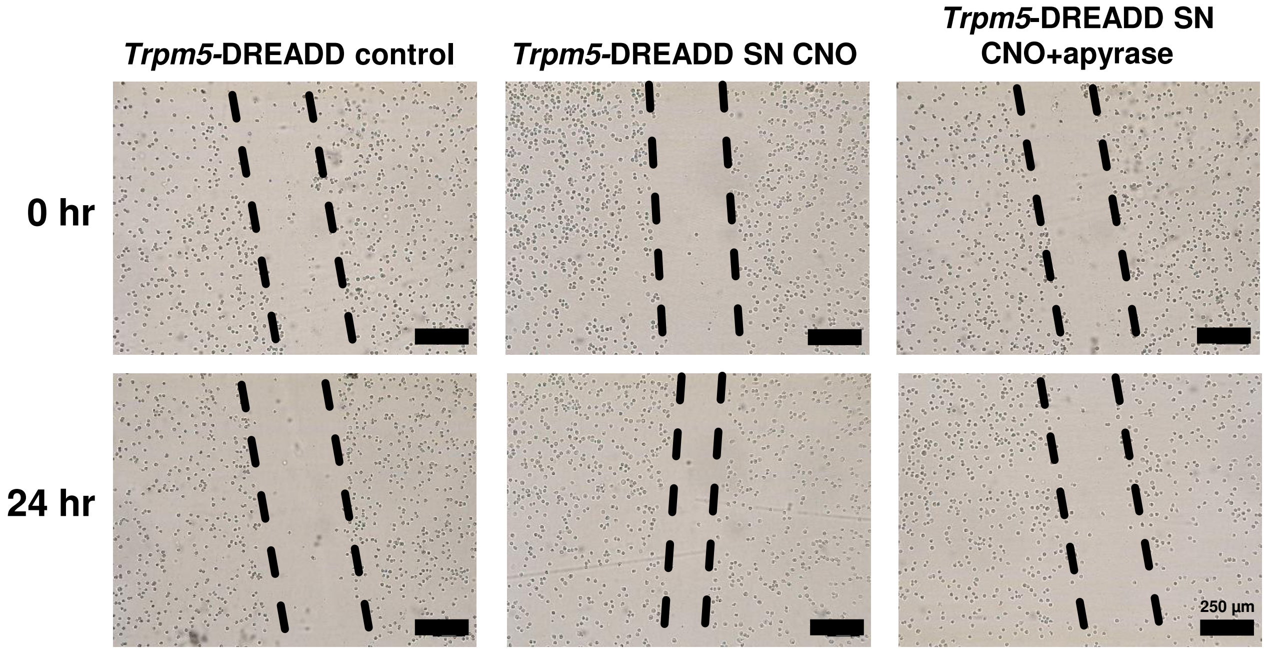

Fig. S21: Migration assay with dendritic cells. Representative images of dendritic cells before (0 h) and after stimulation (24 h) with supernatants (SN) obtained from tracheae of *Trpm5*-DREADD mice treated with vehicle or CNO (100 µM), in the presence or absence apyrase (5 U/ml). Scale bar = 250 µm. The quantitative analyses of the migration assay can be found in Figure 7e. (n=3 independent experiments)
