# Supplementary material for: Tracheal tuft cells release ATP and link innate to adaptive immunity in pneumonia
Source: Nat Commun. 2025 Jan 10;16:584. doi: 10.1038/s41467-025-55936-5 (PMC11724094; doi:10.1038/s41467-025-55936-5)
Supplement: Supplementary file 2 — Reporting Summary [file 41467_2025_55936_MOESM2_ESM.pdf]

nature portfolio

Corresponding author(s): Gabriela Krasteva-Christ
Last updated by author(s): Dec 16, 2024

Reporting Summary

Nature Portfolio wishes to improve the reproducibility of the work that we publish. This form provides structure for consistency and transparency in reporting. For further information on Nature Portfolio policies, see our Editorial Policies and the Editorial Policy Checklist.

Statistics

For all statistical analyses, confirm that the following items are present in the figure legend, table legend, main text, or Methods section.

n/a	Confirmed	
☐	☒	The exact sample size (*n*) for each experimental group/condition, given as a discrete number and unit of measurement
☐	☒	A statement on whether measurements were taken from distinct samples or whether the same sample was measured repeatedly
☐	☒	The statistical test(s) used AND whether they are one- or two-sided<br>*Only common tests should be described solely by name; describe more complex techniques in the Methods section.*
☒	☐	A description of all covariates tested
☐	☒	A description of any assumptions or corrections, such as tests of normality and adjustment for multiple comparisons
☐	☒	A full description of the statistical parameters including central tendency (e.g. means) or other basic estimates (e.g. regression coefficient) AND variation (e.g. standard deviation) or associated estimates of uncertainty (e.g. confidence intervals)
☐	☒	For null hypothesis testing, the test statistic (e.g. *F*, *t*, *r*) with confidence intervals, effect sizes, degrees of freedom and *P* value noted<br>*Give P values as exact values whenever suitable.*
☒	☐	For Bayesian analysis, information on the choice of priors and Markov chain Monte Carlo settings
☒	☐	For hierarchical and complex designs, identification of the appropriate level for tests and full reporting of outcomes
☐	☒	Estimates of effect sizes (e.g. Cohen's *d*, Pearson's *r*), indicating how they were calculated

Our web collection on statistics for biologists contains articles on many of the points above.

Software and code

Policy information about availability of computer code

Data collection	*NIS-Elements version AR 5.10.01 (Nikon Instruments), FlexAnalysis® software version 3.4 (Bruker Daltonics), Zen pro version 2.3 (Zeiss), Patchmaster version v2x90.3 (HEKA), Kaleido 2.0 (Perkin Elmer), LabChart version 8 (ADInstruments), LAS v3.7.0 (Leica Application Suite), FACSuite™ Software version 1.0.6.5230 (BD), NucleoView NC-200 version 1.3.0.0 (Chemomatec), CFX Manager version 3.1 (Bio-Rad), Milliplex Analyst version 3.5 (Merck Millipore)*
Data analysis	*FlexAnalysis® software version 3.4 (Bruker Daltonics), Igor Pro version 8.0.4.2 (WaveMetrics), FlowJo version 10.9.0 (BD Biosciences), Microsoft Office 2013, GraphPad Prism 10.2.2 (GraphPad software Inc.), ImageJ 1.52a (NIH), Image Lab 4.0.1 (Bio-Rad)*

For manuscripts utilizing custom algorithms or software that are central to the research but not yet described in published literature, software must be made available to editors and reviewers. We strongly encourage code deposition in a community repository (e.g. GitHub). See the Nature Portfolio guidelines for submitting code & software for further information.

Data

Policy information about availability of data

All manuscripts must include a data availability statement. This statement should provide the following information, where applicable:

- Accession codes, unique identifiers, or web links for publicly available datasets
- A description of any restrictions on data availability
- For clinical datasets or third party data, please ensure that the statement adheres to our policy

The data generated in this study are included in the Source Data file, which is provided with this paper.

Research involving human participants, their data, or biological material

Policy information about studies with human participants or human data. See also policy information about sex, gender (identity/presentation), and sexual orientation and race, ethnicity and racism.

Reporting on sex and gender	*n/a*
Reporting on race, ethnicity, or other socially relevant groupings	*n/a*
Population characteristics	*n/a*
Recruitment	*n/a*
Ethics oversight	*n/a*

Note that full information on the approval of the study protocol must also be provided in the manuscript.

Field-specific reporting

Please select the one below that is the best fit for your research. If you are not sure, read the appropriate sections before making your selection.

☒ Life sciences ☐ Behavioural & social sciences ☐ Ecological, evolutionary & environmental sciences

For a reference copy of the document with all sections, see nature.com/documents/nr-reporting-summary-flat.pdf

Life sciences study design

All studies must disclose on these points even when the disclosure is negative.

Sample size	Sample size was estimated using PASS 2019 software in consultation with statisticians affiliated with our university.
Data exclusions	All data were included, except for values identified as statistical outliers using the Grubbs test.
Replication	Experiments were independently reproduced at least three times to assess reproducibility.
Randomization	Allocation was random, as mice, cells and samples were selected randomly.
Blinding	During in vivo experiments and those involving freshly isolated epithelial or tuft cells, mouse information was not blinded due to animal protection regulations, which require clear documentation of each mouse from birth to death. However, following sample preparation, data collection and analysis were performed in a blinded manner.

Behavioural & social sciences study design

All studies must disclose on these points even when the disclosure is negative.

Study description	*n/a*
Research sample	*n/a*
Sampling strategy	*n/a*
Data collection	*n/a*
Timing	*n/a*

Data exclusions	*n/a*
Non-participation	*n/a*
Randomization	*n/a*

Ecological, evolutionary & environmental sciences study design

All studies must disclose on these points even when the disclosure is negative.

Study description	*n/a*
Research sample	*n/a*
Sampling strategy	*n/a*
Data collection	*n/a*
Timing and spatial scale	*n/a*
Data exclusions	*n/a*
Reproducibility	*n/a*
Randomization	*n/a*
Blinding	*n/a*

Did the study involve field work? ☐ Yes ☐ No

Field work, collection and transport

Field conditions	*n/a*
Location	*n/a*
Access & import/export	*n/a*
Disturbance	*n/a*

Reporting for specific materials, systems and methods

We require information from authors about some types of materials, experimental systems and methods used in many studies. Here, indicate whether each material, system or method listed is relevant to your study. If you are not sure if a list item applies to your research, read the appropriate section before selecting a response.

Materials & experimental systems

n/a	Involved in the study	
☐	☒	Antibodies
☐	☒	Eukaryotic cell lines
☒	☐	Palaeontology and archaeology
☐	☒	Animals and other organisms
☒	☐	Clinical data
☒	☐	Dual use research of concern
☒	☐	Plants

Methods

n/a	Involved in the study	
☒	☐	ChIP-seq
☐	☒	Flow cytometry
☒	☐	MRI-based neuroimaging

Antibodies

Antibodies used	1. Pannexin 1 (dilution: 1:200, host: rabbit, Alomone labs, Cat.No. ACC-234, polyclonal)<br>2. Trpm5 (dilution 1:800, host: rabbit, created in-house (Hollenhorst et al. 2020)<br>3. DCAMKL1 (dilution 1:1600, host: rabbit, abcam, Cat.No. ab31704, Lot: GR335/375-1, polyclonal)

4. ChAT (dilution 1:300, host: goat, Sigma-Aldrich, Cat.No. AB144P, Lot: 2603400, polyclonal)
5. CD11c (dilution 1:800, host: Armenian hamster, eBioscience, Cat.No. 14-0114-82, Clone: N418)
6. donkey anti-rabbit-Cy3 (dilution 1:1000, Merck Millipore, Cat.No. AP182C, Lot: 3382285, polylonal)
7. donkey anti-goat-Cy3 (dilution 1:250, Merck, Cat.No. AP180C, Lot: 2707848, polyclonal)
8. goat anti-armenian-hamster-Cy3 (dilution 1:800, Jackson, Cat.No. 127-165-160, Lot: 137164, polyclonal)
9. donkey anti-rabbit-Cy2 (dilution 1:100, Jackson, Cat.No. 711-255-152, Lot: 156228, polyclonal)
10. ChAT (dilution 1:500, host: rabbit, ThermoFisher, Cat.No. 703789, Lot: 2367073, Clone: 13H9L16)
11. anti-rabbit-HRP (dilution 1:10000, Sigma-Aldrich, Cat.No. 12-348, polyclonal)
12. F4/80-PE-Cy7 (dilution: 1:40, eBioscience, Cat.No. 25-4801-82, Lot: 2279168, Clone: BM8)
13. CD45-PerCP-Cy5.5 (dilution 1:133, eBioscience, Cat.No. 45-0451-82, Lot: 1994158, Clone: 30-F11)
14. CD11b-FITC (dilution 1:40, eBioscience, Cat.No. 11-0112-82, Lot: 2125442, Clone: M1/70)
15. CD86-PE (dilution 1:133, eBioscience, Cat.No. 12-0862-82, Lot: 2045467, Clone: GL1)
16. CD11c-APC-Cy7 (dilution 1:40, BD Biosciences, Cat.No. 561241, Lot: 0352741, Clone: HL3)
17. CCR6-APC (dilution 1:40, Biolegend, Cat.No. 129814, Lot: B339849, Clone: 29-2L17)
18. CD4-FITC (dilution 1:80, eBioscience, Cat.No. 11-0042-82, Lot: 2235746, Clone: RM4-5)
19. LY6G-APC (dilution 1:40, eBioscience, Cat.No. 17-9668-82, Lot: 2183550, Clone: 1A8-Ly6g)
20. IL-17A-PE-Cy7 (dilution 1:40, eBioscience, Cat.No. 25-7177-82, Lot: 2317033, Clone: eBio17B7)
21. F4/80-FITC (dilution 1:50, BioLegend, Cat.No. 123107, Lot: B361743, Clone: BM8)
22. CD11b-Pacific blue (dilution 1:50, BioLegend, Cat.No. 101223, Lot: B350151, Clone: M1/70)
23. Ep-CAM-PE-Cy7 (dilution 1:50, BioLegend, Cat.No. 118215, Lot: B303317, Clone: G8.8)
24. MHCII-PE-Cy7 (dilution 1:333, BioLegend, Cat.No. 107629, Lot: B322006, Clone: N5/114.15.2)
25. CD86-Pacific blue (dilution 1:50, BioLegend, Cat.No. 105022, Lot: B377787, Clone: GL-1)
26. Gnat3 (dilution 1:400, Covalab, Cat.No. pab73402, Lot: 65974, polyclonal)
27. ChAT (dilution 1:400, Sigma-Aldrich, Cat.No. AB144P, Lot: 2603400, polyclonal)
28. GFP (dilution 1:200, Novusbio, Cat.No. NB100-1614, polyclonal)
29. donkey anti-goat-AF647 (dilution 1:400, Millipore, Cat.No. AP180SA6, Lot: 3743391, polyclonal)
30. donkey anti-chicken-FITC (dilution 1:200, Jackson ImmunoResearch, Cat.No. 703-096-155, Lot: 164518, polyclonal)
31. CD16/32 (dilution 1:100, eBioscience, Cat.No. 14-0161-82, Clone : 93)
32. LY6C-APC (dilution 1:40, BioLegend, Cat.No. 128015, Lot: B322022, Clone: HK1.4)
33. CCR7-PE (dilution 1:20, BioLegend, Cat.No. 120105, Lot: B387617, Clone: 4B12)
34. CD3-eFluor450 (dilution 1:40, eBioscience, Cat.No. 48-0032-82, Lot: 1987699, Clone: 17A2)
35. TCR-APC (dilution 1:80, BioLegend, Cat.No. 109211, Lot: B397293, Clone: H57-597)
36. TCRg/d-FITC (dilution 1:50, BioLegend, Cat.No. 118105, Lot: B401688, Clone: GL3)
37. KLRG-BV421 (dilution 1:20, BioLegend, Cat.No. 138414, Lot: B354054, Clone: 2F1/KLRG1)
38. CD45-APC-Cy7 (dilution 1:40, BioLegend, Cat.No. 103116, Lot: B371499, Clone: 30-F11)
39. CD127-PerCP-Cy5.5 (dilution 1:40, BioLegend, Cat.No. 121114, Lot: B321692, Clone: SB/199)
40. T-bet-APC (dilution 1:40, BioLegend, Cat.No. 644814, Lot: B353938, Clone: 4B10)
41. RORgT-PE (dilution 1:50, BD Biosciences, Cat.No. 562607, Lot: 2040776, Clone: Q31-378)
42. CD5-biotin (dilution 1:200, BioLegend, Cat.No. 100604, Lot: B314434, Clone: 53-7.3)
43. CD3-biotin (dilution 1:200, BioLegend, Cat.No. 100304, Lot: B357833, Clone: 145-2C11)
44. GR-1-biotin (dilution 1:200, BioLegend, Cat.No. 108404, Lot: B357272, Clone: RB6-8C5)
45. CD45R-biotin (dilution 1:200, BioLegend, Cat.No. 103203, Lot: B352761, Clone: RA3-6B2)

Validation

All antibodies used in this study except the Trpm5 antibody are commercially available. Antibodies were purchased from eBioscience, BioLegend, Sigma-Aldrich, Merck, Jackson, Millipore, Merck Millipore, abcam and Alomone labs. All commercial antibodies have been validated as mentioned on the manufacturer's websites listed below. The Trpm5 antibody was validated in tissue from Trpm5-deficient mice (Hollenhorst et al. 2022).
1. https://www.alomone.com/p/anti-pannexin-1/ACC-234
3. https://www.abcam.com/en-de/products/primary-antibodies/dcamkl1-antibody-ab31704
4. https://www.sigmaaldrich.com/DE/en/product/mm/ab144p
5. https://www.thermofisher.com/antibody/product/CD11c-Antibody-clone-N418-Monoclonal/14-0114-82
6. https://www.merckmillipore.com/DE/de/product/Donkey-Anti-Rabbit-IgG-Antibody-Cy3-conjugate-Species-Adsorbed,MM_NF-AP182C
7. https://www.merckmillipore.com/DE/de/product/Donkey-Anti-Goat-IgG-Antibody-Cy3-conjugate-Species-Adsorbed,MM_NF-AP180C?ReferrerURL=https%3A%2F%2Fwww.google.com%2F
8. https://www.jacksonimmuno.com/catalog/products/127-165-160
9. https://www.jacksonimmuno.com/catalog/products/711-225-152
10. https://www.thermofisher.com/antibody/product/ChAT-Antibody-clone-13H9L16-Recombinant-Monoclonal/703789
11. https://www.sigmaaldrich.com/DE/en/product/mm/12348
12. https://www.thermofisher.com/antibody/product/F4-80-Antibody-clone-BM8-Monoclonal/25-4801-82
13. https://www.thermofisher.com/antibody/product/CD45-Antibody-clone-30-F11-Monoclonal/45-0451-82
14. https://www.thermofisher.com/antibody/product/CD11b-Antibody-clone-M1-70-Monoclonal/11-0112-82
15. https://www.thermofisher.com/antibody/product/CD86-B7-2-Antibody-clone-GL1-Monoclonal/12-0862-82
16. https://www.bdbiosciences.com/en-us/products/reagents/flow-cytometry-reagents/research-reagents/single-color-antibodies-ruo/apc-cy-7-hamster-anti-mouse-cd11c.561241
17. https://www.biolegend.com/nl-nl/products/apc-anti-mouse-cd196-ccr6-antibody-6117
18. https://www.thermofisher.com/antibody/product/CD4-Antibody-clone-RM4-5-Monoclonal/11-0042-82

19. https://www.thermofisher.com/antibody/product/Ly-6G-Antibody-clone-1A8-Ly6g-Monoclonal/17-9668-82
20. https://www.thermofisher.com/antibody/product/IL-17A-Antibody-clone-eBio17B7-Monoclonal/25-7177-82
21. https://www.biolegend.com/de-de/products/fitc-anti-mouse-f4-80-antibody-4067?GroupID=BLG5319
22. https://www.biolegend.com/de-de/products/pacific-blue-anti-mouse-human-cd11b-antibody-3863
23. https://www.biolegend.com/de-de/products/pe-cyanine7-anti-mouse-cd326-ep-cam-antibody-5303?GroupID=BLG6455
24. https://www.biolegend.com/de-de/products/pe-cyanine7-anti-mouse-i-a-i-e-antibody-6136?GroupID=BLG11931
25. https://www.biolegend.com/de-at/products/pacific-blue-anti-mouse-cd86-antibody-3122?GroupID=BLG10719
26. https://www.covalab.com/catalog/product/view/_ignore_category/1/id/304640/s/p21920-gnat3-internal-antibody/
27.https://www.sigmaaldrich.com/CY/en/product/mm/ab144p?utm_source=google&utm_medium=cpc&utm_campaign=21473655026&utm_content=160056090610&gclid=EAIaIQobChMIlZOK6-XliAMVl2lBAh0tkRFuEAAYASAAEgIs__D_BwE
28. https://www.novusbio.com/products/gfp-antibody_nb100-1614
29. https://www.merckmillipore.com/INTL/en/search/AP180SA6?search=&TrackingSearchType=SB+-+Search+Box&SearchContextPageletUUID=&SearchTerm=AP180SA6
30. https://www.jacksonimmuno.com/catalog/products/703-096-155
31. https://www.thermofisher.com/antibody/product/CD16-CD32-Antibody-clone-93-Monoclonal/14-0161-82
32. https://www.biolegend.com/de-de/products/apc-anti-mouse-ly-6c-antibody-6047
33. https://www.biolegend.com/en-ie/products/pe-anti-mouse-cd197-ccr7-antibody-2799
34. https://www.thermofisher.com/antibody/product/CD3-Antibody-clone-17A2-Monoclonal/48-0032-82
35. https://www.biolegend.com/de-de/products/apc-anti-mouse-tcr-beta-chain-antibody-268
36. https://www.biolegend.com/nl-be/products/fitc-anti-mouse-tcr-gamma-delta-antibody-2420
37. https://www.biolegend.com/en-gb/products/brilliant-violet-421-anti-mouse-human-klrg1-mafa-antibody-7528?GroupID=BLG8908
38. https://www.biolegend.com/ja-jp/products/apc-cyanine7-anti-mouse-cd45-antibody-2530?GroupID=BLG1932
39. https://punchout.biolegend.com/fr-fr/products/percp-cyanine5-5-anti-mouse-cd127-il-7ralpha-antibody-4517?GroupID=BLG10447
40. https://www.biolegend.com/de-at/products/apc-anti-t-bet-antibody-7120?GroupID=BLG6433
41. https://www.bdbiosciences.com/en-us/products/reagents/flow-cytometry-reagents/research-reagents/single-color-antibodies-ruo/pe-mouse-anti-mouse-ror-t.562607
42. https://www.biolegend.com/en-gb/products/biotin-anti-mouse-cd5-antibody-158
43. https://www.biolegend.com/ja-jp/products/biotin-anti-mouse-cd3epsilon-antibody-22
44. https://www.biolegend.com/ja-jp/products/biotin-anti-mouse-ly-6g-ly-6c-gr-1-antibody-457
45. https://www.biolegend.com/nl-be/products/biotin-anti-mouse-human-cd45r-b220-antibody-444

Eukaryotic cell lines

Policy information about cell lines and Sex and Gender in Research

Cell line source(s)	HEK293 cells were acquired from ATCC (No. CRL-1573)
Authentication	*n/a*
Mycoplasma contamination	*All cells were regularly tested for mycoplasma.*
Commonly misidentified lines (See ICLAC register)	*n/a*

Palaeontology and Archaeology

Specimen provenance	*n/a*
Specimen deposition	*n(a*
Dating methods	*n/a*

☐ Tick this box to confirm that the raw and calibrated dates are available in the paper or in Supplementary Information.

Ethics oversight	*n/a*

Note that full information on the approval of the study protocol must also be provided in the manuscript.

Animals and other research organisms

Policy information about studies involving animals; ARRIVE guidelines recommended for reporting animal research, and Sex and Gender in Research

Laboratory animals	Mice of both genders, older than 8 weeks of age, were used from the following genetic lines: Trpm5-/- (Damak et al. 2006), ChAT-

	eGFP (Tallini et al. 2006), Trpm5-/-/ChAT-eGFP (Tg(RP23-268L19-EGFP)2Mik;Trpm5tm1Dgen(129S5/SvEvBrd-C57BL/6)), Trpm5-DTA (Perniss et al. 2020), Trpm5-DREADD (designer receptors exclusively activated by designer drugs) (Yu et al. 2023), Trpm5-DREADD-tGFP (Yu et al. 2023), Chatfl/flTrpm5cre B6;129-Trpm5tm1.1(cre)Uboe;B6;129-Chattm1Jrs/J, Pannexin1-/- (Panx1-/-) (Anselmi et al. 2008), Pannexin2-/- (Panx2-/-) (Bargiotas et al. 2011) and Pannexin1-/-/2-/- (Panx1-/-/2-/-) (Bargiotas et al. 2011) as well as wild-type controls. Mice of both genders were included in the experiments, and no gender specific effects were studied.
Wild animals	*The study did not involve wild animals.*
Reporting on sex	*There were no sex-based analyses performed.*
Field-collected samples	The study did not involve samples collected from the filed.
Ethics oversight	All animal experimental and care procedures were conducted in accordance with the German guidelines for care and use of laboratory animals as well as the ARRIVE guidelines, and were approved by the Animal Welfare Committee of Saarland (approval numbers 04/2018, 40/2018, 09/2021).

Note that full information on the approval of the study protocol must also be provided in the manuscript.

Clinical data

Policy information about clinical studies

All manuscripts should comply with the ICMJEguidelines for publication of clinical research and a completedCONSORT checklist must be included with all submissions.

Clinical trial registration	*n/a*
Study protocol	*n/a*
Data collection	*n/a*
Outcomes	*n/a*

Dual use research of concern

Policy information about dual use research of concern

Hazards

Could the accidental, deliberate or reckless misuse of agents or technologies generated in the work, or the application of information presented in the manuscript, pose a threat to:

No	Yes	
☒	☐	Public health
☒	☐	National security
☒	☐	Crops and/or livestock
☒	☐	Ecosystems
☒	☐	Any other significant area

Experiments of concern

Does the work involve any of these experiments of concern:

No	Yes	
☒	☐	Demonstrate how to render a vaccine ineffective
☒	☐	Confer resistance to therapeutically useful antibiotics or antiviral agents
☒	☐	Enhance the virulence of a pathogen or render a nonpathogen virulent
☒	☐	Increase transmissibility of a pathogen
☒	☐	Alter the host range of a pathogen
☒	☐	Enable evasion of diagnostic/detection modalities
☒	☐	Enable the weaponization of a biological agent or toxin
☒	☐	Any other potentially harmful combination of experiments and agents

Plants

Seed stocks	*n/a*
Novel plant genotypes	*n/a*
Authentication	*n/a*

ChIP-seq

Data deposition

- [ ] Confirm that both raw and final processed data have been deposited in a public database such as GEO.
- [ ] Confirm that you have deposited or provided access to graph files (e.g. BED files) for the called peaks.

Data access links<br>*May remain private before publication.*	*n/a*
Files in database submission	*n/a*
Genome browser session<br>(e.g. UCSC)	*n/a*

Methodology

Replicates	*n/a*
Sequencing depth	*n/a*
Antibodies	*n/a*
Peak calling parameters	*n/a*
Data quality	*n/a*
Software	*n/a*

Flow Cytometry

Plots

Confirm that:

- [x] The axis labels state the marker and fluorochrome used (e.g. CD4-FITC).
- [x] The axis scales are clearly visible. Include numbers along axes only for bottom left plot of group (a 'group' is an analysis of identical markers).
- [x] All plots are contour plots with outliers or pseudocolor plots.
- [x] A numerical value for number of cells or percentage (with statistics) is provided.

Methodology

Sample preparation	Lungs, tracheae, bronchoalveolar lavage fluid (BALF) and airways lymph nodes were collected from CNO- or denatonium-treated, P. aeruginosa-infected mice or untreated control mice of each mouse strain used, and processed as described previously (Hollenhorst et al, 2022). Briefly, euthanasia (see above), blood samples were collected followed by obtaining BALF through instillation and aspiration of 1 ml of ice-cold DPBS containing 0.1 mM EDTA and 1% fetal bovine serum (FBS) via a tracheal cannula into the lungs. The last step was repeated three times. The mice were then perfused with cold DPBS, and the tracheae, lungs and airway lymph nodes were carefully dissected. For single-cell suspension preparation, tissues were finally cut into small pieces. Lungs were digested for 1 h at 37°C using a solution of collagenase II (1 mg/ml, Gibco) and DNase I (1 µl/ml, Invitrogen) while tracheas were digested for 30 min with solution containing papain (20 U/ml, Sigma-Aldrich), EDTA (1.6 mM, Grüssing), L-Cysteine (25 mM, Sigma-Aldrich) and DNase I (0.5 µl/ml, Invitrogen) . Airway lymph nodes were mechanically disrupted without enzymatic digestion. Subsequently, all tissues, except blood, were filtered through a 70 µm cell strainer to obtain single cells (Falcon, BD Biosciences, Heidelberg, Germany). Red blood cells (RBCs) were lysed using RBCs

	ACK lysis buffer (Thermo Fisher Scientific). Cell counts and viability were assessed using NucleoCounter® NC-200™ (Chemomatec, Kaiserslautern, Germany). For CNO-treated mice, cell viability was assessed using the Zombie Aqua Fixable Viability Kit (1:100, BioLegend, San Diego, California, USA) following the manufacturer's protocol. Finally, cells were fixed using 1% PFA (or 2% PFA for samples from CNO-treated animals).
Instrument	The data were collected using a BD FACSverse (BD Biosciences). For the isolation of the dendritic cells for culture, either a SH800S cell sorter (Sony biotechnology, San Jose, CA, USA) or a FACS Aria III (BD Biosciences) was used..
Software	The data were analyzed using BD FACSuite™ Software (BD Biosciences).
Cell population abundance	The purity of sorted dendritic cells (DCs) was 80%, as determined by reanalyzing the sorted samples.
Gating strategy	The gating strategy for DCs was: CD45+ LY6G- F4/80- CD11b+ CD11c+.

☒ Tick this box to confirm that a figure exemplifying the gating strategy is provided in the Supplementary Information.

Magnetic resonance imaging

Experimental design

Design type	*n/a*
Design specifications	*n/a*
Behavioral performance measures	*n/a*

Acquisition

Imaging type(s)	*n/a*
Field strength	*n/a*
Sequence & imaging parameters	*n/a*
Area of acquisition	*n/a*

Diffusion MRI ☐ Used ☐ Not used

Preprocessing

Preprocessing software	*n/a*
Normalization	*n/a*
Normalization template	*n/a*
Noise and artifact removal	*n/a*
Volume censoring	*n/a*

Statistical modeling & inference

Model type and settings	*n/a*
Effect(s) tested	*n/a*

Specify type of analysis: ☐ Whole brain ☐ ROI-based ☐ Both

Statistic type for inference (See Eklund et al. 2016)	*n/a*
Correction	*n/a*

Models & analysis

n/a	Involved in the study
☐	☐ Functional and/or effective connectivity
☐	☐ Graph analysis
☐	☐ Multivariate modeling or predictive analysis

Functional and/or effective connectivity	*n/a*
Graph analysis	*n/a*
Multivariate modeling and predictive analysis	*n/a*
